# The Chemistry of Plant–Microbe Interactions in the Rhizosphere and the Potential for Metabolomics to Reveal Signaling Related to Defense Priming and Induced Systemic Resistance

**DOI:** 10.3389/fpls.2018.00112

**Published:** 2018-02-09

**Authors:** Msizi I. Mhlongo, Lizelle A. Piater, Ntakadzeni E. Madala, Nico Labuschagne, Ian A. Dubery

**Affiliations:** ^1^Department of Biochemistry, University of Johannesburg, Johannesburg, South Africa; ^2^Department of Plant and Soil Sciences, University of Pretoria, Pretoria, South Africa

**Keywords:** chemical communication, induced resistance, metabolites, metabolomics, plant–microbe interactions, priming, signalomics

## Abstract

Plant roots communicate with microbes in a sophisticated manner through chemical communication within the rhizosphere, thereby leading to biofilm formation of beneficial microbes and, in the case of plant growth-promoting rhizomicrobes/-bacteria (PGPR), resulting in priming of defense, or induced resistance in the plant host. The knowledge of plant–plant and plant–microbe interactions have been greatly extended over recent years; however, the chemical communication leading to priming is far from being well understood. Furthermore, linkage between below- and above-ground plant physiological processes adds to the complexity. In metabolomics studies, the main aim is to profile and annotate all exo- and endo-metabolites in a biological system that drive and participate in physiological processes. Recent advances in this field has enabled researchers to analyze 100s of compounds in one sample over a short time period. Here, from a metabolomics viewpoint, we review the interactions within the rhizosphere and subsequent above-ground ‘signalomics’, and emphasize the contributions that mass spectrometric-based metabolomic approaches can bring to the study of plant-beneficial – and priming events.

## Introduction: Sustainable Production of Food Plants

The world is facing a concerning challenge to produce sufficient food in a sustainable manner, with an increasing global population and decreasing food resources. Food plant production is hampered by a plethora of biotic stresses such as pathogens and herbivores (Iriti and Faoro, 2009; Gust et al., 2010; Thakur and Sohal, 2013). To defend themselves, plants rely on innate immunity of which the success in fighting disease infections or herbivore feeding depends on how rapid and strong an activated immune can be deployed. To combat plant diseases and limit the use of pesticides and herbivore agrochemicals, genetic modification has been used (Bhandari, 2014). However, the use of such strategies has caused major debates citing environmental – (Aktar et al., 2009; Bhandari, 2014) and consumer concerns (Ferreira et al., 2012); hence, the need for new eco-friendly strategies. In the context of plant protection, priming refers to a stimulus or treatment for improved responses to upcoming environmental challenges. Colonization of plant roots by beneficial microbes in the rhizosphere is such a stimulus since it may result in ISR which have a positive effect on the ability of the plant to defend itself against attack by pathogens infecting the leaves (Hilker et al., 2015). Here, we highlight chemical communication in the rhizosphere (plant roots interacting with plant-beneficial rhizobacteria and – fungi) and ISR or RMPP as an environmentally friendly method to combat pathogens and herbivores, as investigated through the use of LC coupled to MS-based metabolomics.

## Pre-Formed Barriers and Plant Immune Responses: Potential Obstacles for Interactions with Rhizomicrobes

**FIGURE 1:**
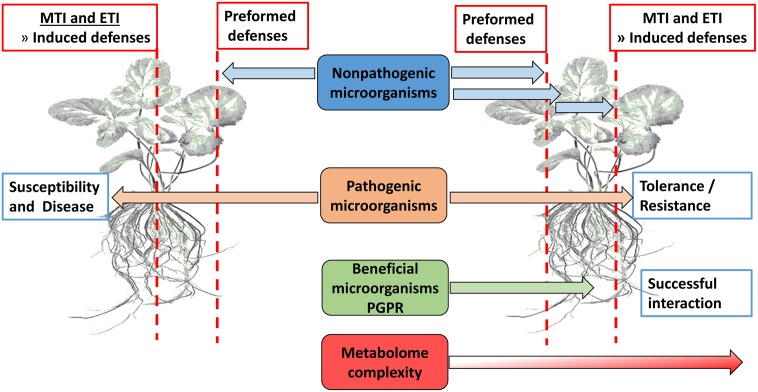
Overview of physical barriers (waxes, suberin, callose, lignin, etc.) and innate immunity defenses (MTI and ETI) that presents obstacles to potential microorganisms in establishing a beneficial interaction with plant roots.

Plants use preformed defense mechanisms aimed at preventing both pathogen entrance and herbivore feeding (**Figure 1**). Failure hereof, either below- or above-ground (De Coninck et al., 2015), leads to plant activation of an immune response termed microbe/pathogen-associated molecular pattern (MAMP)-triggered immunity (MTI) which relies on the detection of conserved microbial signature molecules (MAMPs) *via* extracellular transmembrane receptors or PRRs (Jones and Dangl, 2006; Conrath et al., 2009; Sanabria et al., 2009; Deslandes and Rivas, 2012; Denancé et al., 2013; Gao et al., 2013). Some pathogens are capable of down-regulating MTI by the secretion of effector molecules, thereby leading to effector-triggered susceptibility (ETS). To overcome this, plant resistance (R) proteins recognize these molecules and activate a second line of defense which is a rapid and robust response termed ETI (Pieterse et al., 2009; Gao et al., 2013; De Coninck et al., 2015), and which is associated with the hypersensitive response (HR). The MTI and ETI sections of induced immunity are complementary, and signaling interactions occur between MTI and ETI at very early stages. Furthermore, MTI and ETI share many biochemical features, but differ in the intensity or amplitude of the host responses (Zipfel, 2008; Zhang and Zhou, 2010; Dempsey and Klessig, 2012). Damage-associated molecular patterns (DAMPs) are molecules arising from necrotic, damaged or stressed cells, e.g., cutin monomers, small peptides, and cell wall fragments. Plants recognize these molecules in a similar manner as MAMPs and respond by activating defense signaling cascades (Herman et al., 2008; Yamaguchi et al., 2010; Liu et al., 2013; De Coninck et al., 2015). These plant defense responses are strictly regulated in order to minimize resource expenditure and fine-tune the signaling cascades. This crucial role is fulfilled by phytohormones like SA, JA, and ET as essential signaling molecules (Bartoli et al., 2013) for both local and systemic responses. It is important to note that this basic signaling defense is more complex because of other phytohormones including ABA, auxins, cytokinins, gibberellins, and brassinosteroids (BRs) that interplay in the background. Recently, even more plant signaling molecules such as azelaic acid (AZA), pipecolic acid (PIP), and strigolactones have been reported (Pieterse et al., 2009; Denancé et al., 2013; Vos et al., 2013). In order to establish an effective symbiotic relationship between plants and PGPR, these preformed barriers and innate immunity defenses have to be bypassed through chemical communication between plant and microbe (**Figure 1**).

Recent findings indicate that symbionts and pathogens deploy similar molecular strategies to dampen and overcome immune responses, and that the MAMP/PRR recognition system is also engaged in cooperative plant-microbe interactions with beneficial microbial communities that can lead to root colonization. This suggests a multifaceted management role by microbial communities of the innate immune system for controlled accommodation of beneficial microbes *vs.* pathogen elimination (Hacquard et al., 2017).

## Chemical Communication Within the Rhizosphere

The rhizosphere is one of the most complex ecosystems on earth and is inhabited by various organisms including nematodes, fungi, bacteria, and arthropod herbivores (Venturi and Keel, 2016). Compared to bulk soil, the rhizosphere is associated with increased bacterial abundance and activity, but lower diversity. Plants are known to effect a selective pressure on the microbial community found in the rhizosphere and community-level analysis have revealed differential microbial communities associated with different plant species. This suggests a definite role of plant-derived metabolites in the microbiome assemblage in the rhizosphere (Hacquard et al., 2017; Yang et al., 2017; Zhang et al., 2017). The common PGPR genera in the rhizosphere includes: *Bacillus*, *Pseudomonas*, *Enterobacter*, *Acinetobacter*, *Burkholderia*, *Arthrobacter*, and *Paenibacillus* (Finkel et al., 2017; Sasse et al., 2017; Zhang et al., 2017).

Recent knowledge advancement in plant-beneficial microbe interactions has led to the development and commercialization of microbial inoculation (either one or a consortium) to improve plant health. These inoculants are natural or synthetic microbial communities (Johns et al., 2016). This is done in one of the following ways: (1) introduction of new microbes into the soil, (2) manipulation of environmental factors (temperature, nutrients, moisture level, etc.), and (3) growing plants that will influence the soil microbe community (Finkel et al., 2017; Pineda et al., 2017).

**FIGURE 2:**
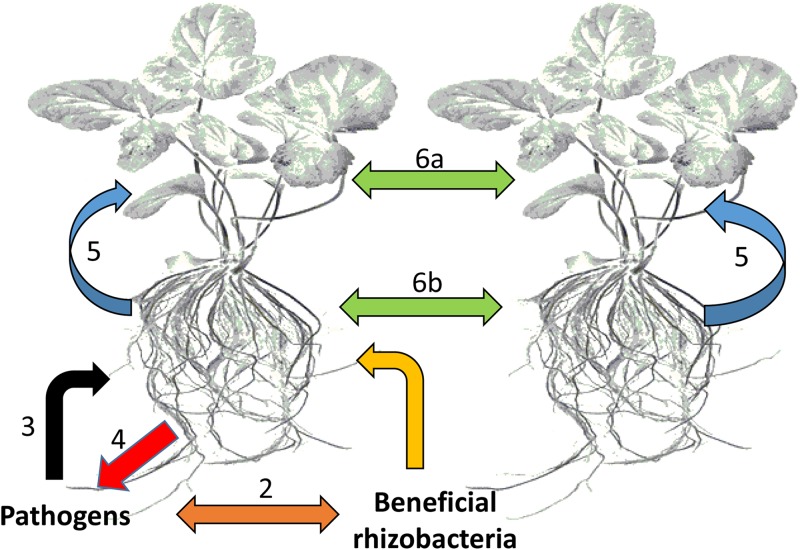
Types of chemical-based interactions affecting plants. The plant host plus all its symbiotic microbes can be regarded as a community or an ecological unit (holobiont). (1) Interactions between plant roots and beneficial bacteria within the rhizosphere, (2) competitive interactions between beneficial bacteria and potential pathogens, (3) attack by potential pathogens on plant roots, (4) counter defense responses against pathogen attack, (5) communication between plants roots and leaves, and (6) interplant communication through leaves (6a) and roots (6b).

**FIGURE 3:**
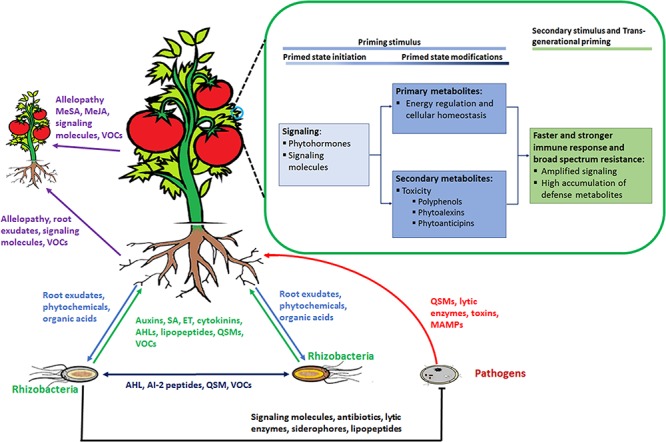
Rhizosphere plant and microbial ‘signalomics’. Plants and rhizomicrobes secrete compounds beneficial to each other to establish mutual relationships. This below–ground interaction, in turn, primes plants against various environmental stimuli that includes abiotic as well as biotic stresses. Perception of the priming stimulus leads to activation of signaling molecules, primary metabolism regulation and gene activation of enzymes involved in the production of secondary defense metabolites. When a secondary stimulus is detected the same process as in the priming stage takes place but at an enhanced level to minimize impact on the plant. Plants are able to pass on the induced primed state to their progeny in a process known as *trans*-generational priming. In addition, plants communicate with each other using allelopathic molecules. Abbreviations: volatile organic compounds (VOCs), quorum sensing molecules (QSM), *N*-acyl homoserine lactones (AHL), SA, methylsalicylic acid (MeSA), methyljasmonic acid (MeJA) ET.

These organisms interact with each other and with the plant in a sophisticated manner, achieved by chemical communication established in the rhizosphere (**Figure 2**). In response to altered gene expression, plants subsequently release an array of metabolites (primary and secondary). It is through such communication that mutual relationships are established that are vital for root–root interactions (Mommer et al., 2016), nutrient availability, microorganism accumulation, and biofilm formation of soil microbial communities (Rosier et al., 2016; Sasse et al., 2017), as well as inhibition of soil–borne pathogens (Bertin et al., 2003; Li et al., 2013). In this regard, metabolomic approaches have enabled researchers to identify and quantify compounds secreted by the microorganisms as well as profiling the metabolite ‘blends’ present in root exudates that play a vital role in this mutual interaction (**Figure 3**). The term ‘signalomics’ describes these metabolomics approaches employed to decipher the chemical communications occurring within the rhizosphere.

### Bacteria-to-Bacteria Communication

Soil bacteria present in the microbiome assemblages produce an array of signaling metabolites that affect gene expression within the host plants, and these compounds have become an important and interesting subject for researchers. Here, VOCs are the well-documented signaling molecules within bacterial communities (**Figure 3**). These are low-molecular weight lipophilic compounds synthesized from different metabolic pathways and serve as a chemical window in which information is released (Kanchiswamy et al., 2015). Recently is has been shown that VOCs play a greater role in microbial communication than the non-volatile counterparts. Rhizobacteria produce numerous VOCs comprising alkanes, alkenes, alcohols, ketones, terpenoids and sulfur compounds. Furthermore, the metabolite complexity of the volatile profiles is attributed to species – or genotype – specific metabolism (Kanchiswamy et al., 2015; Tyc et al., 2015; Kai et al., 2016).

Colonization of plant roots by PGPR involve QS, a cell-to-cell communication mechanism through the release of signals to cognate receptors, thereby influencing gene expression in correlation to bacterial population density (González and Marketon, 2003; Hong et al., 2012; Helman and Chernin, 2015). These signals, also referred to as autoinducers, allows both intra- and inter-bacterial communication between different species (González and Marketon, 2003; Hassan et al., 2016).

### Bacteria-to-Plant Communication

To establish a symbiotic relation with plants, rhizobacteria either secrete or emit molecules beneficial to the plant. These molecules, originating from the rhizosphere, are able to trigger specific changes or adjustments to the plant transcriptome. While phytohormones are growth – and defense regulators produced by plants, PGPR are also able to produce these compounds that include auxins, cytokinins, gibberellins, ABA, SA, and JA, among others (**Figure 3**) (Fahad et al., 2015). VOCs produced by PGPR are involved in maintaining soil health, plant growth modulation and resistance induction (Wei-wei et al., 2008; Kai et al., 2009). Certain plants are responsive toward various known VOCs produced by PGPR such as 2-heptanol, 2-endecanone, and pentadecane. For example, co-cultivation of *Arabidopsis thaliana* and two PGPR strains (*Bacillus subtilis* GB03 and *B. amyloliquefaciens* IN937a) in Petri dishes (allowing diffusion of bacterial volatiles from one side to another) resulted in enhanced growth of *A. thaliana*. Here, 3-hydroxy-2-butanone (acetoin) and 2,3 butanediol were the common VOCs between the two strains (Ryu et al., 2003).

Quorum sensing is a population density mechanism used by bacterial communities to communicate and sense their environment. AHLs are the well documented QS signals frequently produced by Gram-negative bacteria (González and Marketon, 2003; Hassan et al., 2016). AHLs are perceived by plants and contribute to the establishment of a bacterial–plant symbiotic relationship (Schikora, 2016). UHPLC-MS (ultra-high performance liquid chromatography coupled to MS) methodology as described in the Section “Metabolomics: A Tool for Analysis of Plant Interactions with Rhizomicrobes” can precisely detect and quantify AHLs as well as the *N*-acyl homoserine degradation products, thereby enabling the study of signaling dynamics in QS (Rothballer et al., 2018).

### Plant-to-Bacteria Communication

The chemical complexity of root exudates is dependent on a number of external factors such photosynthesis activity, plant size, and soil conditions. These secreted metabolites (**Figure 3**) are species- or genotype-specific and can be differentially modified depending on the secreting source. Given this strong complexity and specificity, root exudates have the potential to overlay a much more detailed layer of information about the communication events in the rhizosphere (Mommer et al., 2016; Sasse et al., 2017). Also, the chemical compositions of root exudates have a direct effect on the rhizosphere communities and it has been shown that specific plant species use these compounds to select soil microbe communities. For example, citric acid identified from cucumber root exudates attracted *B. amyloliquefaciens* SQR9 and cause biofilm formation. In addition, the banana root exudate fumaric acid attracted *B. subtilis* N11 and stimulated biofilm formation (Zhang et al., 2014). Studies have also shown that strain growth and antifungal activity of certain *Pseudomonas* spp. is dependent on organic acids and sugars isolated from tomato root exudates (Kravchenko et al., 2003).

Another class of compounds found in the root exudates are flavonoids (i.e., 2 phenyl-1,4-benzopyrone derivatives) which induce bacterial *nod* genes, thus leading to lipo-chitooligosaccharides (LCOs) that initiate nodule formation in the roots. Interestingly, LCO also plays a role in interactions between arbuscular mycorrhizal fungi and plants. Furthermore, these flavonoids are able to mimic bacterial QS molecules, thus influencing bacterial metabolism (Hassan and Mathesius, 2012). QS plays an important role in bacterial genotype and phenotype regulation for successful root colonization (Rosier et al., 2016). Different types of low carbon molecules are also present in the root exudates; these molecules serve as precursors for biosynthesis of PGPR phytohormones. Tryptophan, which is a precursor for indole-3-acetic acid, is concentrated in the root tip region (Haichar et al., 2014). In addition, the ET precursor, aminocyclopropane-1-carboxylic acid (ACC), also exudes from plants and can be used as a source of nitrogen and carbon by PGPR (Haichar et al., 2012).

### Plant-to-Plant Communication

Communication between plants occurs below- and above-ground (**Figure 2**), either through secretion/release of certain signaling molecules (Mommer et al., 2016). Root exudates released into the rhizosphere contain a blend of signaling molecules that are transmitted to neighboring plants (Badri and Vivanco, 2009). However, root–root interactions are mostly studied in the context of species competition and invasive plants. Allelopathy is the most prominent probability, in which plants release phytotoxins such as catechin (a flavan-3-ol flavonoid). This compound is able to mediate intraspecific and interspecific interactions, and to inhibit establishment and growth of neighboring plants, thus reducing competition and increasing nutrient availability (Thorpe et al., 2009; Mommer et al., 2016). Compounds with allelopathic effects belong to one of the following chemical classes: benzene-derived compounds, phenolics, hydroxamic acids, and terpenes (Badri and Vivanco, 2009; Massalha et al., 2017). On the other hand, VOCs are the most studied allelochemicals in plant-plant interactions. VOC-mediated signaling in the rhizosphere is believed to occur through common mycorrhizal networks between plants, protecting them against degradation and enhancing plant-to-plant transmission. Beside rhizosphere signaling, plants do secrete their own VOCs into the air that are carried to neighboring plants (**Figure 3**).

## Rhizosphere Defense and Prime-Inducing Compounds

Over the years, PGPR have been extensively studied for plant growth promotion and ISR induction, and are promising alternatives to chemical fertilization, pesticides, and herbicides (Kloepper et al., 2004; Gupta et al., 2015). PGPR effect beneficial properties through direct mechanisms (i.e., nitrogen fixation, mineral solubilization and biosynthesis of phytohormone and siderophore production) and indirect mechanisms (production of antibiotics, hydrolytic enzymes, siderophores, LPs, and ISR) (Beneduzi et al., 2012; Garcia-Fraile et al., 2015; Gupta et al., 2015). Here, we look at the major classes of molecules secreted by PGPR that are involved in plant protection against soil-borne pathogens and induction of ISR/RMPP.

Antibiotics and related molecules are secreted by certain bacteria and have the ability to inhibit pathogen growth at low concentrations (**Figure 3**). Such compounds from *Bacillus* and *Pseudomonas* genera are the best studied in disease management (Haas and Défago, 2005; Saha et al., 2012). For example, 2,4 diacetylphloroglucinol (2,4 DAPG) is an antibiotic produced by *P. fluorescens* that has a 75% inhibition effectiveness against the soil-borne pathogen *Sclerotium rolfsii* (Asadhi et al., 2013). Phenazine-1-carboxylic acid (PCA) is another antimicrobial compound secreted by the same organism and causes oxidation-reduction and accumulation of superoxides in target cells. This molecule is effective against wheat disease caused by *Gaeumannomyces graminis* var. *tritici* and *S. rolfsii*, causing stem rot in groundnut (Lohitha et al., 2016). Another novel antibiotic from *B. subtilis* is zwittermicin which is effective against a spectrum of soil-borne pathogens (Saraf et al., 2014). Several bacteria secrete hydrolytic enzymes, e.g., proteases, glucanases, chitinases, lipases and amylases. These enzymes degrade numerous cell wall components of fungi and oomycetes (Bull et al., 2002; Saraf et al., 2005, 2014).

Various PGPR such as *Bacillus* spp. and others, produce LPs (either linear or cyclic LPs) that act as antibiotics. These are classified into three families: iritin, fengycin and surfactin depending on the branching fatty acid (Saha et al., 2012; Saraf et al., 2014), and have antagonistic effects against a wide range of soil-borne pathogens. Besides being antagonistic to pathogens, LPs such as fengycin, surfactin and iturin are capable of inducing immune responses in plants by acting as bacterial determinants (Ongena et al., 2007; Romero et al., 2007). The role of these molecules in ISR/RMPP has been studied on various plants. In bean and tomato both purified and compounds from producing strains were found to induce immune responses or prime plants (Ongena et al., 2007). *B. subtilis* S499 can prime cucumber plants against *Colletotrichum lagenarium*. However, plants treated with semi-purified LPs were susceptible to *C. lagenarium* (Ongena et al., 2005a). Recent studies on LPs involvement in immune responses strongly show that these molecules are involved in ISR or RMPP. For example, cyclic LPs purified from *B. amyloliquefaciens* subsp. *plantarum*, isolated from the lettuce rhizosphere, primed plants against *Rhizoctonia solani* (Chowdhury et al., 2015).

Siderophores are low molecular weight compounds synthesized by microorganisms under iron limiting conditions. With high membrane permeability, siderophores act as ferric ion transport vehicles into microbial cells (Butler and Theisen, 2010). The common iron-binding substances in these compounds include hydroxamic acid, hydrocarboxylic acid, and catechols, as well as other related structures (Pattus and Abdallah, 2000; Butler and Theisen, 2010; Ahmed and Holmström, 2014). Siderophore production is beneficial to plants (directly supply iron to plants) and is implicated in soil-borne disease suppression (reducing competitiveness of soil-borne pathogens) (Tank et al., 2012). A mutant of *P. putita* over-expressing siderophores was more effective against *Fusarium* wilt in tomato when compared to a siderophore-deficient mutant of *P. aeruginosa* which lost its biocontrol ability. Furthermore, *B. subtilis*-produced siderophores exhibit antagonistic effects against wilt and dry root rot- causing fungi in chickpea (Patil et al., 2014). Also, purified siderophores had similar disease suppression activity to those observed from the producing strains.

Several reports have demonstrated that AHLs can influence plant physiological processes such as root elongation (Bai et al., 2012), plant perception (Han et al., 2016), and induce a broad spectrum resistance (Schikora, 2016). Plant priming by AHLs has recently been documented with reports that even commercial available pure AHLs also induce priming in plants (Schenk et al., 2014). For examples, both short and long chain AHLs produced by *Serratia liquefaciens* strain MG1 and *P. putida* strain IsoF primed tomato plants against *A. alternata* via SA and ET defense pathway (Schuhegger et al., 2006). Also, in barley endophytic *Acidovorax radicis* N35 rhizobacteria producing 3-hydroxy-decanoyl-homoserine lactone induced defense responses and caused accumulation of flavonoids such as saponarin and lutonarin (Han et al., 2016).

Among the metabolites produced by PGPR, volatiles are small molecules that can effectively promote plant growth, induce resistance and inhibit growth of pathogenic organisms (Ryu et al., 2004; Beneduzi et al., 2012; Song and Ryu, 2013). For example, volatiles emitted by different rhizobacterial isolates were reported to inhibit mycelial growth of *Rhizoctonia solani* (Kai et al., 2007). High vapor pressure volatiles are able to diffuse in the soil (Insam and Seewald, 2010), which gives these compounds an advantage to act at distance. *In vitro*, volatiles from four *Bacillus* and *Paenibacillus* spp. showed intensive antagonistic activities against soil-borne pathogens including *Ascochyta cutrillina*, *Alternarai solani*, and *A. brassicae*. From GC-head space analysis, four metabolites namely 2,4 decadienal, oleic acid, diethyl phthalate, and *n*-hexadecanoic acid showed overlapping presence among the strains (Wei-wei et al., 2008).

Plants rapidly recognize both potential pathogens and PGPR (**Figure 1**) in a similar manner based on MAMPs such as lipopolysaccharide (LPS) and flagellin, and secondary metabolites. MAMPs from beneficial microbes are known to activate MTI, but in this case, the activated defenses do not ward off the beneficial microbes (Van Wees et al., 2008). This is not fully understood, but might involve the nature of the complex chemical communication involved in rhizobacteria-plant interactions. As mentioned, PGPR produce plant signaling molecules such as auxins, cytokinins, gibberellins, ABA, SA, ET, and JA (Fahad et al., 2015). It is well known that SA, ET, and JA cross-communicate to fine-tune the defense response, depending on the detected stimulus (Dempsey and Klessig, 2012; Derksen et al., 2013). SA is an interesting signaling molecule produced by certain PGPR. For example, several *Pseudomonas* spp. produce SA under low iron conditions which is channeled toward SA-containing siderophores (Mercado-Blanco and Bakker, 2007). However, SA produced by *P. aeruginosa* (siderophore producing mutant KMPCH) was shown to induce systemic resistance (Audenaert et al., 2002; Verhagen et al., 2010). Thus, both MAMPs and SA are involved in RMPP.

In response to different stimuli, plants emit numerous VOCs with signaling and inhibitory properties. These within-plant VOCs signaling leads to induction and priming of plant defense (Heil and Silva Bueno, 2007). Among other volatiles profiled in head-space experiments, MeSA, MeJA, and *cis*-jasmone (CJ) are well documented volatile signaling molecules (Heil and Silva Bueno, 2007). These were found to induce plant defense and priming against herbivore-feeding in wild lima beans. CJ has been tested on various plants and it has been shown to induce production of defense-related VOCs such as (*E*)-ocimene, 6-methyl-5-hepten-2-one and (*E*)-(*1R,9S*)-caryophyllene (Pickett et al., 2007). Also, (*Z*)-3-hexen-1-ol was found to have a two-fold priming effect and modulation of herbivorous insect behavior (Wei and Kang, 2011). Even though progress has been made in understanding the involvement of plant VOCs in signaling, attraction of predators and pathogen inhibition, there is no knowledge on plant VOCs induced in response to ISR/PGPR-priming by rhizomicrobes. However, metabolomic studies have shown that regardless of the perceived stimulus, similar metabolic pathways are activated (Pastor et al., 2014; Balmer et al., 2015; Mhlongo et al., 2016a,b). Thus, such studies suggest that the blend of signaling VOCs is the same/similar, leading to production of defense metabolites within the producing plant as well as in distal plants.

## ISR/Rhizomicrobe Plant Priming (RMPP)

In the rhizosphere, a complex relationship exists among plants, soil microbes, and soil (Van Dam and Bouwmeester, 2016). The microbial diversity (population and activity) in this zone is influenced by physical, chemical and biological properties of the root-associated soil (Barea et al., 2002). The rhizosphere is inhabited by both deleterious and beneficial microbes (**Figure 2**) that can significantly influence plant growth and crop yield (Beneduzi et al., 2012; Vacheron et al., 2013; Garcia-Fraile et al., 2015). The beneficial microbes include symbiotic bacteria, free-living bacteria, actinomycetes, and mycorrhizal fungi that increase nutrients/plant growth enhancer availability and suppress soil-borne pathogens (Garcia-Fraile et al., 2015). Diverse genera of PGPR dominated by *Bacillus* and *Pseudomonas* spp. have been identified, and are the most desirable beneficial group for their variable qualities such as plant growth promotion, disease control and bioremediation. The mechanisms utilized by PGPR to suppress diseases and herbivores as well as priming of plants, have been critically studied and reviewed over the last few years (Saraf et al., 2005, 2014; Pineda et al., 2010; Beneduzi et al., 2012). PGPR may either directly (inhibition of metabolism) or indirectly (through competition) reduce soil-borne pathogen infections. Some PGPR such as *Bacillus* and *Pseudomonas* spp. synthesize antibiotics that are active against various bacterial and fungal pathogens, toxins against insect pests, lytic enzymes that inhibit soil-borne pathogen growth, and siderophores. Production of cyanogenic compounds have also been shown to repel both root and leaf herbivores. Lastly, PGPR present in the rhizosphere may prevent plant diseases by competing for available nutrients, preventing contact between the pathogen and the plant root, or by interfering with the mechanisms leading to plant infection (Saraf et al., 2005, 2014).

The concept of plant priming dates back to 1901 when Beauverie and Ray showed that plants infected by a pathogen developed an enhanced defense response against secondary infections. This lead to the realization that plants can be sensitized/primed to produce an enhanced defense response, thereby making the plants more resistant to secondary environmental stresses. While it is evident that plant defense can be induced and may lead to less resource expenditure (reduced fitness cost), the success depends on the appropriate activation of defenses that can be faster, earlier, more sensitive, or stronger. These timeous activation of suitable defense responses in primed plants can save plants from becoming diseased or consumed, thus adding a benefit of off-set the cost of establishing the primed condition (Conrath et al., 2009, 2015; Tanou et al., 2012; Hilker et al., 2015).

Studies using PGPR have identified genes associated with ISR/RMPP. For example, transcriptome analysis of *P*. *fluorescens* WCS417r-ISR hosting plants showed systemic expression of defense genes when compared to the control, and *P. syringae* infection led to identification of genes (mostly JA- and ET-regulated genes) with more enhanced expression than non-ISR expressing plants (Bakker et al., 2007; Van Wees et al., 2008; Conrath et al., 2009; Segarra et al., 2009; Vacheron et al., 2013). Also, ISR/RMPP can be induced by PGPR volatiles without the organisms being in contact with the roots. *Bacillus* spp. producing volatiles such as 3-hydroxy-2-butanone and (2R,3R)-(-)-2,3-butanediol were found to prime *Arabidopsis* plants against pathogen infections and herbivore attack (Conrath et al., 2001; Farag et al., 2013; Song and Ryu, 2013; Yi et al., 2013).

Priming can also be a result of epigenetic changes from small interfering RNA (siRNA) or DNA recombination caused by environmental stresses (Bruce et al., 2007; Pastor et al., 2012). This form of protection is present in the genetic material of the species and would last longer in plants compared to accumulation of metabolites. Since plants are not capable of communication with their progeny, a mechanism is required to alert against possible stresses that may be encountered in nature (Holeski et al., 2012). It was not until the early 1980s when *trans*-generational studies were conducted showing that inoculation of a plant with a disease-causing agent induces resistance in their progeny not only to the administered agent, but to a wide spectrum of pathogens (Pieterse, 2012; Slaughter et al., 2012). In addition, other studies showed that plants that have been infected by a pathogen produce seeds with higher levels of phytoalexins than controls. Epigenetic changes or *trans*-generational priming can be inherited by the progeny, where it then controls expression of defense genes (Holeski et al., 2012). In a study where *Arabidopsis* plants were primed with β-aminobutyric acid (BABA) or by MAMPs from *P. syringae*, the progeny showed high levels of defense gene expression *via* the SA-dependent pathway and was resistant to *P. syringae* and *Hyaloperonospora arabidopsidis.* These progenies also had a stronger priming phenotype than the parents. *Trans*-generational priming is achieved through defense response memorization and propagation (in both meiosis and mitosis) by the parents (Luna et al., 2012; Pastor et al., 2012; Slaughter et al., 2012; Po-Wen et al., 2013). However, since there are many mechanisms associated with priming and research aiming at these are still underway, it is not clear how this memorization occurs. The involvement of chromatin modifications adds to the other metabolite-based mechanisms since it is directly linked to gene expression patterns that can be inherited by the offspring.

## Key Metabolic Events in Defense Priming

The priming ability of PGPR is associated with cell wall modification, expression of defense genes, primary metabolite modification and biosynthesis of secondary metabolites (Conrath et al., 2009). As shown in **Figure 3**, priming can be divided in to three major events: (1) perception of the priming stimulus, (2) secondary stimulus, and (3) *trans*-generational priming. The early stages of priming involve signaling by phytohormones and other signaling molecules. Phytohormones are well-documented plant metabolites involved in different stages (**Figure 3**) of plant defense responses or plant priming (Dempsey and Klessig, 2012; Denancé et al., 2013). For example, JA and ET are major hormones in ISR/PGPR priming induction, while SA is the major hormone involved in systemic acquired resistance (SAR). Other phytohormones such as cytokinins, auxins, ABA, gibberellins, and brassinosteroids are reported to play a role in plant resistance but the significance of these molecules is not well understood (De Vos et al., 2005; Koornneef and Pieterse, 2008; Pieterse et al., 2009; Naseem and Dandekar, 2012; Denancé et al., 2013; Uhrig et al., 2013). These hormones interact either antagonistically or synergistically with the SA-JA-ET signaling backbone and reprogram the defense output (Koornneef and Pieterse, 2008; Verhage et al., 2010; Naseem and Dandekar, 2012).

Using *P. fluorescens* as inducer, a total of 50 metabolites were differentially regulated in ISR-induced *Arabidopsis* plants. Amongst these, amino acids and sugars were the differentiated primary metabolites (van de Mortel et al., 2012). ISR/PGPR priming studies are mostly based on molecular rather than metabolomics approaches. Hence, knowledge about metabolome changes during ISR/PGPR priming and the significance thereof, is limited. However, the metabolic events in priming in response to chemical elicitation are more similar, despite the use of different stimuli (Pastor et al., 2014; Balmer et al., 2015; Mhlongo et al., 2016a,b). As such, metabolic studies employing other agents may be used to explain the role of both primary and secondary metabolites in plant priming (Djami-Tchatchou et al., 2017).

The main role of primary metabolism during plant defense is to supply energy for the initiation of plant priming and in the synthesis/activation of phytohormones, phytoanticipins, and phytoalexins. Here, the energy referred to is required for different processes such as defense gene expression of various defense pathways, plant metabolism regulation and resource re-channeling toward defense. As a result, plant priming responses are associated with minor fitness costs when compared to naïve plants. Thus, priming activation leads to temporal down regulation of other metabolic pathways. Recently it has been shown that both signaling molecules (Mhlongo et al., 2017) and secondary metabolite conjugates accumulated during the priming stage (Mhlongo et al., 2016a,b), and can be converted to their active forms when a secondary stress is detected. Glycosylated signaling molecules, specifically that of AZA, SA, and MeSA, were found to accumulate during LPS-induced priming of tobacco cells (Mhlongo et al., 2017). Also, glycosylation of hydroxycinnamic acids was observed in tobacco cells treated with both chemical and pathogen-derived priming agents (Mhlongo et al., 2016a,b). Besides sugar conjugation, the respiratory cycle and tricarboxylic acid cycle (TCA) are also affected by priming activation (Gamir et al., 2014). In this regard, TCA intermediates (citrate, malate, 2-oxalate) were found to over-accumulate in BABA-induced priming. Furthermore, amino acids serve as building blocks for many secondary metabolites such as SA, polyamines, tyramine, alkaloids, and phenylpropanoids.

Secondary metabolites play an important role in plant defense systems and environmental adaptation, and their presence fluctuates in response to different environmental stimuli (Dörnenburg, 2004). As discussed above, PGPR are able to trigger secondary metabolism by means of different chemical molecules. Many studies have shown that mycorrhizal or rhizobacterial root colonization quantitatively modify phenolic compounds, alkaloids, terpenoids, and essential oils in plants (Toussaint et al., 2007; Araim et al., 2009; Ramos-Solano et al., 2015). Using nine PGPR strains on blackberry plants, Ramos-Solano et al. (2015) showed that phenolics, flavonoids, and anthocyanins were the modified secondary metabolites associated with delayed post-harvest fungal growth on berries. Other secondary metabolites such as coumarins and flavonoids also quantitatively changed in plants associated with PGPR (van de Mortel et al., 2012). In maize significant changes in benzoxaninones were observed in plants associated with mycorrhizal or rhizobacterial colonization (Song et al., 2011). Also, maize root inoculation with *P. putita* KT2440 induced metabolic changes and systemic resistance in the plants. The early responses were *via* JA- and ABA-dependent pathways, and phospholipids were highlighted as the important metabolites in the KT2440 interaction. Lastly, benzoxaninones were differentially abundant in roots after 3 days (Planchamp et al., 2014).

Microbial compounds such as LPs and AHLs can also prime plants through modification of secondary metabolites (Ongena et al., 2005a; Schenk et al., 2014; Chowdhury et al., 2015; Han et al., 2016). LP-overproducing *Bacillus* activated the lipoxygenase enzyme (LOX) regulated pathway (Blée, 2002). In potato tuber cells, fengycin treatments resulted in activation of phenylpropanoid pathway metabolism (Ongena et al., 2005b). Moreover, AHLs stimulated callose deposition and accumulation phenolics, oxylipins and SA in several plant species (Schenk et al., 2014; Schikora, 2016).

Plants are capable of maintaining the primed state throughout their life cycle and passing it on to the next generation (*trans*-generational priming) (**Figure 3**) (Luna et al., 2012; Pieterse, 2012; Munné-Bosch and Alegre, 2013; Mauch-Mani et al., 2017). Epigenetic modification is the well-documented *trans*-generational priming mechanism (Gamir et al., 2014; Mauch-Mani et al., 2017). The few reports available on metabolomics related to *trans*-generational priming suggest that phytohormone levels are not modified in the progeny of primed plants (Luna et al., 2012). However, Mandal et al. (2012) showed that progeny resistant to tobacco mosaic virus (TMV) had enhanced levels of primary metabolites, particularly sucrose, glucose, and fructose and the amino acids; ala, val, ser, thr, gln. Despite the lack of documented metabolomic work describing *trans*-generational priming, Gamir et al. (2014) suggested that this process is highly dependent on the characteristics of the pathogen. For example, biotrophic stimuli mainly impact primary metabolism while insects and necrotrophic fungi trigger secondary metabolism *via* JA/ET-dependent pathways.

## Metabolomics: a Tool for Analysis of Plant Interactions with Rhizomicrobes

**FIGURE 4:**
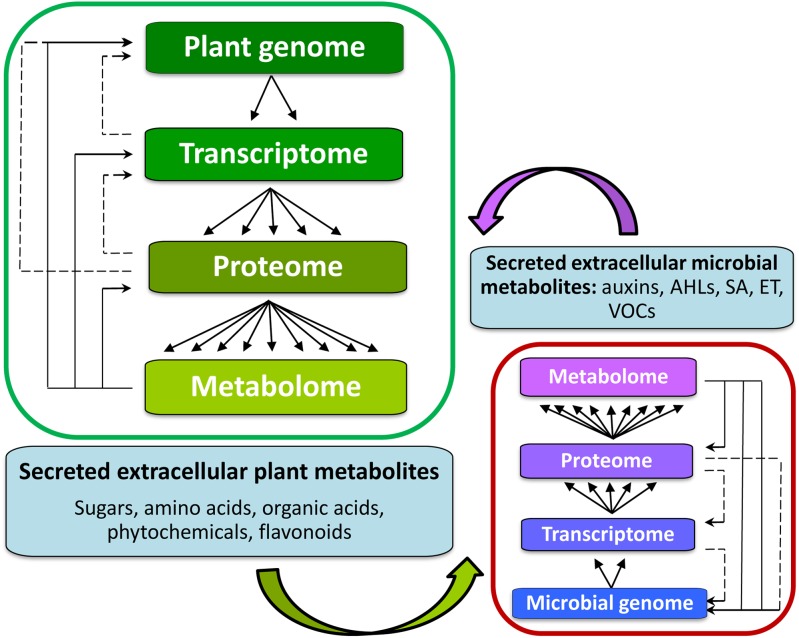
Background for metabolomics studies of signaling in the rhizosphere between plant hosts **(left)** and microorganisms within the rhizosphere **(right)**. The inter-organismal communication affects the biological information flow from genome to metabolome. The metabolome is complementary to the transcriptome and proteome, captures the functional, or physiological state of the cell, and provides a communications link between genotype and phenotype. Metabolites also form part of the regulatory systems in an integrated manner (solid lines indicating regulatory loops). Altered gene expression is ultimately reflected in changes in the pattern and/or concentration of metabolites. It is through these interactions amongst the members of the central dogma components, that a cell acquires its full functionality of its cellular metabolism.

Metabolomics, an array of advanced bio-analytical techniques in conjunction with chemometrics and bioinformatics tools, enables characterization of the perturbations to the metabolomes of interacting organisms (Tugizimana et al., 2013) (**Figure 4**). As stated, the rhizosphere can contain a spectrum of different microbial communities, constituting very complex chemical environments. Metabolomics, as a data-driven, hypothesis-generating scientific approach with the aim to detect and quantify 100s of compounds per analysis (Lloyd et al., 2015), is ideally suited to the analysis of complex interactions and promises to facilitate the modeling of reciprocal responses between plants and organisms within the rhizosphere.

Conceptually, and following a reductionist approach, the tritrophic interaction between plant, rhizomicrobe, and pathogen can be studied separately and in isolation. For example, a co-culture metabolomics approach has been proposed (Allwood et al., 2010) to assess the intracellular metabolomes (metabolic fingerprints) of both host and pathogen and their extruded (extracellular) metabolites (metabolic footprints). However, in order to fully evaluate the changes occurring in the host plant due to these tritrophic interactions under conditions relevant to disease and resistance, there is a need for combining the information provided by different techniques, including metagenomics and metametabolomics (Heinken and Thiele, 2015; Jorge et al., 2016; Ofaim et al., 2017). This novel approach to metabolomics analyses of host–pathogen interactions will facilitate a greater understanding of both their independent metabolism and the metabolic cross-talk which represents the interactome.

**FIGURE 5:**
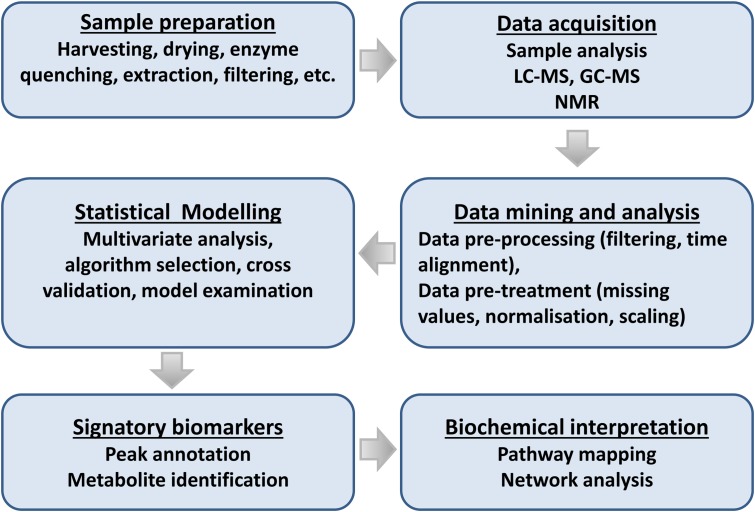
Flowchart for plant metabolomic studies. The three main steps of a metabolomic analysis are sample preparation, data acquisition, and data mining. These three steps are interrelated and lead to the discovery of signatory biomarkers, metabolite annotation, and biochemical interpretation.

Recent advances on both analytical instrumentation and – analysis with high selectivity, accuracy, and robustness, and combined with data processing software developments and availability of public databases, have facilitated this endeavor. Thus, these progressions have enabled researchers not only to study one aspect of a biological system, but also the interaction with the surroundings (Rochfort, 2005; Lloyd et al., 2015; Tenenboim and Brotman, 2016; Van Dam and Bouwmeester, 2016). Below we summarize the main events in an adaptable metabolomics workflow suitable for the study of plant–microbe interactions and highlight some analytical advances (**Figure 5**).

### Sample Preparation

The sample preparation method(s), to a large extent, determines the type of compounds to be detected. Sample preparation for any metabolomics study comprises several steps mostly dictated by the chosen analytical platform. The main steps include material harvesting at a specific time and quenching to minimize metabolic turnover rates. Next, metabolite extraction with organic solvents or solid phase extraction is performed, taking the matrix in which the metabolites occur into account. This is followed by pre-analytical sample preparation (concentration, purification or derivatization) (Tugizimana et al., 2013; Jorge et al., 2016).

### Separation and Detection

Gas chromatography, liquid chromatography (LC), and capillary electrophoreses (CE) coupled to MS have developed into the preferred bio-analytical platforms used in metabolomics (Naz, 2014). GC-MS is usually coupled to a quadrupole (Q), qTOF, and QqQ mass analyzers. In recent years, TOF analyzer interest has grown due to the ability to provide high mass accuracy, higher duty cycles and fast data acquisition in comparison to Q analyzers (Lei et al., 2011; Jorge et al., 2016). QqQ analyzers enable easy compound identification and quantification, and overcome analyte co-elution due to the ability to perform multiple reaction monitoring (MRM) (Gomez-Gonzalez et al., 2010; Lei et al., 2011; Dzier et al., 2012). Recently, GC-MS analysis using stable isotope probing (SIP) has enabled the elucidation of rate limiting steps in metabolic pathways (You et al., 2014). The innovation of GCxGC, using two different stationary phases, provides high separation efficiency and peak capacity, and the generated narrow peaks require a fast scanning mass analyzer such as TOF or semi-fasts scan Q (Adahchour et al., 2005; Jin et al., 2015). One major setback of GC-MS is that it only analyses volatile and thermally stable compounds. To overcome this, derivatization (chemical modification) of molecules with -OH, -COOH, -NH, and -SH functional groups by silylation reagents is employed (Villas-Bôas et al., 2011; Abbiss et al., 2015).

LC-MS column chemistry selection and retention mechanisms makes it the technique most used to complement GC-MS. Most LC-MS applications use reverse phase (RP) and normal phase (NP) stationary phases with eluates eluted with a mobile phase mixture (e.g., organic solvents and water) (Haggarty and Burgess, 2017). Other column chemistries include hydrophilic interaction (HI), ion-exchange (IE), and porous graphitic carbon (PGC) (West et al., 2010). Advances in column dimension and particle size (core-shell and monolithic) has enabled researchers to analyze a wide range of different analytes with high separation efficiency at a high speed (Sanchez et al., 2013; Hayes et al., 2014; Preti, 2016; Urio and Masini, 2015). These column developments lead to the expansion of ultra-high performance liquid chromatography (UHPLC). This chromatography format is similar to HPLC except that it uses a column with particle size ≤2 μm, small column diameter (1–1.2 mm) and operates at high pressure (Sanchez et al., 2013; Fekete et al., 2014; Walter and Andrews, 2014). Electron spray ionization (ESI) is the most popular ionization method preferred in biochemical analysis. This is because it is a soft ionization technique with little internal energy, thus allowing accurate mass determination. Alternatively, by increasing the collision energy, fragmentation can be obtained leading to structural information (Hird et al., 2014; Madala et al., 2014; Ncube et al., 2014). Collision-induced dissociation (CID) with inert gases (He, Ar, or N_2_) is used to obtain more structural information and this is referred to as tandem MS^n^ experiments (Nizkorodov et al., 2011; Madala et al., 2014). Tandem MS^n^ instruments either perform tandem MS^s^ in-time [Ion Trap, Orbitrap, Fourier-transform-ion cyclotron resonance MS (FT-ICR-MS) or in-space (qTOF and QqQ)]. In-time refers to the ability to perform multiple stages of MS achieved by allowing ions from the ion source into the ion trap followed by fragmentation to generate diagnostic information. On the other hand, in-space refers to instruments with two mass analyzers separated by a collision cell which allows two MS stages (Hird et al., 2014).

CE separates compounds based on charge and size, and offers high resolving power. CE-MS is mainly used for intermediate primary metabolic pathways (glycolysis, tricarboxylic acid (TCA) cycle, and pentose phosphate pathway) and is usually coupled to a TOF mass analyzer (Ramautar et al., 2015, 2016).

In recent years, MS imaging (MSI) has been advanced and applied in different metabolic studies. MSI is a new imaging technique that provides the distribution of compounds on the surface (cells, tissue, or specific sections). Here, a two or three dimensional image is created by taking measurements across an individual pixel basis (Wheatcraft et al., 2014; Heyman and Dubery, 2016; Rao et al., 2016). Compared to other traditional molecular imaging techniques, MSI allows a greater amount of information to be obtained by providing well-resolved feature distribution for a wide range of metabolites (Schwamborn, 2012). MSI techniques are divided into non-ambient and ambient approaches. Non-ambient approaches such as matrix assisted laser desorption ionization (MALDI) MS (Heyman and Dubery, 2016) and TOF secondary ion MS (TOF SIMS) have high sensitivity and spatial resolution (Fletcher et al., 2013; Park et al., 2015). These approaches are, however, time-consuming due to the extensive sample preparation and may introduce errors (Park et al., 2015; Heyman and Dubery, 2016; Rao et al., 2016). On the other hand, ambient approaches (desorption electrospray ionization (DESI) MSI, laser ablation electrospray ionization (LAESI) MSI, air-flow-assisted desorption electrospray ionization (AFADESI)-MSI, and nano-DESI MSI), requires less sample preparation and thus produce images of a native state. However, this native state analysis comes with low sensitivity and resolution compared to non-ambient approaches (He et al., 2015; Rao et al., 2016). Recently, single cell analysis (SCA), also referred to as single cell MS (SCMS), has received more attention due to the ability to provide chemical composition of biological samples at cellular level. SCA uses non-ambient, ambient and direct extraction (live single-cell video MS) approaches (Fujii et al., 2015; Onjiko et al., 2015; Rao et al., 2016).

### Data Analysis and Visualization

Metabolomics generates large amounts of complex datasets that require both storage and data processing tools (reduction of data complexity) (Okazaki and Saito, 2012; Berg et al., 2013; Tugizimana et al., 2014). This can be achieved by using free statistical tools such as MarVis1, Mzine, XCMS, MAVEN, Metaboanalyst, MetAlign (Benton et al., 2008) as well as commercial software such as Markerlynx (Waters), Profiling solutions (Shimadzu), Mass profiler pro (Agilent) and Metabolic profiler (Bruker). Such tools focus on homogenous information generation for further statistical analysis. Each calculated *m/z* ion is defined by the same variables that only correspond to it. Recently, Kuich et al. (2015) developed a software (Maui-VIA) specifically for GC-MS data processing. The second step of data analysis involves the application of multivariate statistical tools to reduce data dimensionality, variables discrimination and to reveal shared features among samples (sample clustering). The widely used chemometric methods are unsupervised clustering [principal component analysis (PCA)] and supervised [orthogonal projection to latent structures discriminant analysis (OPLS-DA)] (Trivedi and Iles, 2012; Worley and Powers, 2013).

### Metabolite Annotation and Identification

Metabolite identification is the ultimate goal of any untargeted metabolomics study. Over the years, databases incorporating mass spectra, compound names and structures, statistical models and metabolic pathways have been developed. Such databases complement each other, however, a restricting factor is that the information is scattered and limited by the number of identified metabolites (Fukushima and Kusano, 2013; Sakurai et al., 2013, 2014). Recently, a number of databases incorporating MS or nuclear magnetic resonance (NMR)-based metabolomics and statistical tools have been developed, i.e., MeRy-B, MeltDB, and SetupX (Ferry-Dumazet et al., 2011; Fukushima and Kusano, 2013). Also, a number of integrated databases (e.g., plantmetabolomics.org) are also emerging (Bais et al., 2010). These include full annotation of metabolites, metabolic profiling and statistical tools. This indicates that integrated databases will facilitate metabolomic developments and advances in biological systems.

### Metabolomics Data Storage and ‘Omics’ Data Integration

Initiatives for metabolic data production, storage, dissemination, and analysis to encourage data sharing among researchers have been attempted. MetaboLights is an open access database that contains data, including meta- and raw data, from GC-MS and LC-MS published metabolomics work (Steinbeck et al., 2012).

An integrative study is driven by two purposes: (1) gene function prediction, and (2) systemic interaction characterization of biological systems (Rochfort, 2005; Fukushima et al., 2009; Fernie and Stitt, 2012). ‘-Omics’ analysis produces enormous data sets describing cellular components, their interaction and state of biological networks. Thus, computational methods are needed to reduce this dimension across the wide spectrum of ‘-omics’ data (Blazier and Papin, 2012; Conesa and Herna, 2014). Metabolic network construction is an advantageous platform for ‘-omics’ data integration. It is a manually curated, computational framework that explains gene–protein reaction relationships, assembled from annotated genomes, biochemical reactions, and cell phenotypes (Herrgård et al., 2006; Blazier and Papin, 2012). Thus, to systematically investigate complex host–microbial interactions, a systems biology approach is required that integrates high-throughput data and computational network models. For example, Heinken and Thiele (2015) proposed a constraint-based modeling and analysis approach, that enables the prediction of mechanisms behind metabolic host-microbe interactions on the molecular level.

## Conclusion and Outlook

Recent studies have highlighted the complexity of the rhizosphere as an interlinked ecosystem consisting of different microorganisms that can enhance plant growth through different mechanisms. Chemical communication plays an important role in establishing a mutual relationship between plant roots and PGPR. In addition, both plants and PGPR determine the community of PGPR found in the rhizosphere. In attempts to unravel rhizosphere signalomics, several metabolites, both primary and secondary, have been identified to be the major messengers between plant roots and PGPR. Here, root exudates and PGPR metabolites (non-volatile and volatile) play major roles in establishing a mutual relationship. PGPR are also capable of interfering with phytohormone-linked signaling to inhibit or limit defense responses. PGPR do not only enhance plant growth, but also prime plants against infection by different phytopathogens. ISR/PGPR priming is a result of the complex rhizosphere interaction between plant roots and PGPR, leading to pre-conditioning of plants for an enhanced defense response against secondary stimuli. Most studies done on ISR/PGPR priming are gene- or transcription-based with very few on metabolomics. However, the limited studies available suggest that the early stages involve biosynthesis of signaling molecules followed by modulation of both primary - and secondary metabolism. When secondary stimuli are subsequently perceived, triggered events occur in an enhanced manner. These different physiological states (naïve, primed and primed and triggered) are reflected in changes to the metabolomes and can be investigated through targeted and untargeted metabolomics approaches. However, such studies generally focus on single organisms rather than studying the more complex system consisting of plant, rhizomicrobes and pathogen. Through increased technological advances, both biologically and chemically, we are now better able to study in detail the chemical changes which are associated with microbe-plant interactions and the biochemical mechanisms behind them. Metametabolomics, targeted at the phytobiome would therefore be a future approach aimed at unraveling the complexity of chemical communication in the rhizosphere.

## Author Contributions

Conceived and designed the research: MM and ID. Contributed to the paper and revised it critically for important intellectual content: MM, LP, NM, NL, and ID. All authors gave approval to the final version.

## Conflict of Interest Statement

The authors declare that the research was conducted in the absence of any commercial or financial relationships that could be construed as a potential conflict of interest.

## Abbreviations

ABA: Abscisic acid
AHL: *N*-acyl-homoserine lactone
ET: Ethylene
ETI: Effector-triggered immunity
GC-MS: Gas chromatography mass spectrometry
ISR: Induced systemic resistance
JA: Jasmonic acid
LC-MS: Liquid chromatography mass spectrometry
MAMP: Microbe-associated molecular pattern
MeJA: Methyl jasmonic acid
MeSA: Methyl salicylic acid
MTI: MAMP-triggered immunity
LP: Lipopeptide
PGPR: Plant growth-promoting rhizo-microbes/-bacteria
PRR: Plant pattern recognition receptors
QqQ: Triple quadrupole
QS: Quorum sensing
qTOF: Quadrupole time-of-flight
RMPP: Rhizomicrobe-induced plant priming
SA: Salicylic acid
TOF: Time-of-flight
UHPLC-MS: Ultra-high performance liquid chromatography coupled to mass spectrometry
VOC: Volatile organic compound.

## References

[B1] Abbiss H. Rawlinson C. Maker G. L. Trengove R. (2015). Assessment of automated trimethylsilyl derivatization protocols for GC-MS-based untargeted metabolomic analysis of urine. *Metabolomics* 11 1908–1921. 10.1007/s11306-015-0839-y

[B2] Adahchour M. Brandt M. Baier H. Vreuls J. J. Batenburg A. M. Brinkman U. A. T. (2005). Comprehensive two-dimensional gas chromatography coupled to a rapid-scanning quadrupole mass spectrometer: principles and applications. *J. Chromatogr. A* 1067 245–254. 10.1016/j.chroma.2004.09.094 15844530

[B3] Ahmed E. Holmström S. J. M. (2014). Siderophores in environmental research: roles and applications. *Microb. Biotechnol.* 7 196–208. 10.1111/1751-7915.12117 24576157 PMC3992016

[B4] Aktar W. Sengupta D. Chowdhury A. (2009). Impact of pesticides use in agriculture: their benefits and hazards. *Interdiscip. Toxicol.* 2 1–12. 10.2478/v10102-009-0001-7 21217838 PMC2984095

[B5] Allwood J. W. Clarke A. Goodacre R. Mur L. A. J. (2010). Dual metabolomics: a novel approach to understanding plant–pathogen interactions. *Phytochemistry* 71 590–597. 10.1016/j.phytochem.2010.01.006 20138320

[B6] Araim G. Saleem A. Arnason John T. Charest C. (2009). Root colonization by an Arbuscular Mycorrhizal (AM) fungus increases growth and secondary metabolism of purple coneflower, *Echinacea purpurea* (L.) Moench. *J. Agric. Food Chem.* 57 2255–2258. 10.1021/jf803173x 19239187

[B7] Asadhi S. Reddy Bhaskara B. V. Sivaprasad Y. Prathyusha M. Krishna T. M. Krishna Kumar K. V. (2013). Characterisation, genetic diversity and antagonistic potential of 2,4-diacetylphloroglucinol producing *Pseudomonas fluorescens* isolates in groundnut-based cropping systems of Andhra Pradesh, India. *Arch Phytopathol. Plant Prot.* 45 1966–1977. 10.1080/03235408.2013.782223

[B8] Audenaert K. Pattery T. Cornelis P. Höfte M. (2002). Induction of systemic resistance to *Botrytis cinerea* in tomato by *Pseudomonas aeruginosa* 7NSK2: role of salicylic acid, pyochelin, and pyocyanin. *Mol. Plant. Microbe Interact.* 15 1147–1156. 10.1094/MPMI.2002.15.11.1147 12423020

[B9] Badri D. V. Vivanco J. M. (2009). Regulation and function of root exudates. *Plant Cell Environ.* 32 666–681. 10.1111/j.1365-3040.2009.01926.x 19143988

[B10] Bai X. Todd C. D. Desikan R. Yang Y. Hu X. (2012). N-3-oxo-decanoyl-L-homoserine-lactone activates auxins-induced adventitious roots formation via hydrogen peroxide- and nitric oxide dependent cyclic formation gmp signaling in mug bean. *Plant Physiol.* 158 725–736. 10.1104/pp.111.185769 22138973 PMC3271762

[B11] Bais P. Moon S. M. He K. Leitao R. Dreher K. Walk T. (2010). PlantMetabolomics.org: a web portal for plant metabolomics experiments. *Plant Physiol.* 152 1807–1816. 10.1104/pp.109.151027 20147492 PMC2850039

[B12] Bakker P. A. H. M. Pieterse C. M. J. van Loon L. C. (2007). Induced systemic resistance by fluorescent *Pseudomonas* spp. *Phytopathology* 97 239–243. 10.1094/PHYTO-97-2-0239 18944381

[B13] Balmer A. Pastor V. Gamir J. Flors V. Mauch-Mani B. (2015). The “prime-ome”: towards a holistic approach to priming. *Trends Plant Sci.* 20 443–452. 10.1016/j.tplants.2015.04.002 25921921

[B14] Barea J.-M. Azcon R. Azcon-Aguilar C. (2002). Mycorrhizosphere interactions to improve plant fitness and soil quality. *Antonie Van Leeuwenhoek* 8 343–351. 10.1023/A:1020588701325 12448732

[B15] Bartoli C. C. Casalongué C. A. Simontacchi M. Marquez-Garcia B. Foyer C. H. (2013). Interactions between hormone and redox signalling pathways in the control of growth and cross tolerance to stress. *Environ. Exp. Bot.* 94 73–88. 10.1016/j.envexpbot.2012.05.003

[B16] Beneduzi A. Ambrosini A. Passaglia L. M. P. (2012). Plant growth-promoting rhizobacteria (PGPR): their potential as antagonists and biocontrol agents. *Genet. Mol. Biol.* 4 1044–1051. 10.1590/S1415-47572012000600020 23411488 PMC3571425

[B17] Benton H. P. Wong D. M. Trauger S. A. Siuzdak G. (2008). XCMS2: processing tandem mass spectrometry data for metabolite identification and structural characterization. *Anal. Chem.* 80 6382–6389. 10.1021/ac800795f 18627180 PMC2728033

[B18] Berg M. Vanaerschot M. Jankevics A. Cuypers B. Breitling R. Dujardin J. (2013). LC-MS metabolomics from study design to data-analysis-using a versatile pathogen as a test case. *Comput. Struct. Biotechnol. J.* 4 e201301002. 10.5936/csbj.201301002 24688684 PMC3962178

[B19] Bertin C. Yang X. Weston L. A. (2003). The role of root exudates and allelochemicals in the rhizosphere. *Plant Soil* 256 67–83. 10.1023/A:1026290508166

[B20] Bhandari G. (2014). An overview of agrochemicals and their effects on environment in nepal. *Appl. Ecol. Environ. Sci.* 2 66–73. 10.12691/aees-2-2-5

[B21] Blazier A. S. Papin J. A. (2012). Integration of expression data in genome-scale metabolic network reconstructions. *Front. Physiol.* 3:299 10.3389/fphys.2012.00299 PMC3429070 22934050

[B22] Blée E. (2002). Impact of phyto-oxylipins in plant defense. *Trends Plant Sci.* 7 315–322. 10.1007/s00253-005-1940-3 12119169

[B23] Bruce T. J. A. Matthes M. C. Napier J. A. Pickett J. A. (2007). Stressful “memories” of plants: evidence and possible mechanisms. *Plant Sci.* 173 603–608. 10.1016/j.plantsci.2007.09.002

[B24] Bull C. T. Shetty K. G. Subbarao K. V. (2002). Interactions between myxobacteria, plant pathogenic fungi, and biocontrol agents. *Plant Dis.* 86 889–896. 10.1094/PDIS.2002.86.8.889 30818644

[B25] Butler A. Theisen R. M. (2010). Iron(III)-siderophore coordination chemistry: reactivity of marine siderophores. *Coord. Chem. Rev.* 254 288–296. 10.1016/j.ccr.2009.09.010 21442004 PMC3062850

[B26] Chowdhury S. P. Uhl J. Grosch R. Alquéres S. Pittroff S. Dietel K. (2015). Cyclic lipopeptides of *Bacillus amyloliquefaciens* subsp. *plantarum* colonizing the lettuce rhizosphere enhance plant defense responses toward the bottom rot pathogen *Rhizoctonia solani*. *Mol. Plant Microbe Interact.* 28 984–995. 10.1094/MPMI-03-15-0066-R 26011557

[B27] Conesa A. Herna R. (2014). Omics data integration in systems biology: methods and applications. *Compr. Anal. Chem.* 64 441–459. 10.1016/B978-0-444-62650-9.00016-6

[B28] Conrath P. G. U. Beckers G. J. M. Flors V. García-agustín P. Jakab G. Mauch F. (2009). Priming: getting ready for battle. *Mol. Plant Microbe Interact.* 19 1062–1071. 10.1094/MPMI-19-1062 17022170

[B29] Conrath U. Beckers G. J. M. Langenbach C. J. G. Jaskiewicz M. R. (2015). Priming for ehanced defense. *Annu. Rev. Phytopathol.* 53 97–119. 10.1146/annurev-phyto-080614-120132 26070330

[B30] Conrath U. Thulke O. Katz V. Schwindling S. Kohler A. (2001). Priming as a mechanism in induced systemic resistance of plants. *Eur. J. Plant Pathol.* 107 113–119. 10.1007/s12088-007-0054-2 23100680 PMC3450033

[B31] De Coninck B. Timmermans P. Vos C. Cammue B. P. A. Kazan K. (2015). What lies beneath: belowground defense strategies in plants. *Trends Plant Sci.* 20 91–101. 10.1016/j.tplants.2014.09.007 25307784

[B32] De Vos M. Oosten V. R. Van Poecke R. M. P. Van Pelt J. A. Van Pozo M. J. Mueller M. J. (2005). Signal signature and transcriptome changes of Arabidopsis during pathogen and insect attack. *Mol. Plant Microbe Interact.* 18 923–937. 10.1094/MPMI-18-0923 16167763

[B33] Dempsey D. A. Klessig D. F. (2012). SOS-too many signals for systemic acquired resistance? *Trends Plant Sci.* 17 538–545. 10.1016/j.tplants.2012.05.011 22749315

[B34] Denancé N. Sánchez-Vallet A. Goffner D. Molina A. (2013). Disease resistance or growth: the role of plant hormones in balancing immune responses and fitness costs. *Front. Plant Sci.* 4:155. 10.3389/fpls.2013.00155 23745126 PMC3662895

[B35] Derksen H. Rampitsch C. Daayf F. (2013). Signaling cross-talk in plant disease resistance. *Plant Sci.* 207 79–87. 10.1016/j.plantsci.2013.03.004 23602102

[B36] Deslandes L. Rivas S. (2012). Catch me if you can: bacterial effectors and plant targets. *Trends Plant Sci.* 17 644–655. 10.1016/j.tplants.2012.06.011 22796464

[B37] Djami-Tchatchou A. T. Ncube E. N. Steenkamp P. A. Dubery I. A. (2017). Similar, but different: structurally related azelaic acid and hexanoic acid trigger differential metabolomic and transcriptomic responses in tobacco cells. *BMC Plant Biol.* 17:227. 10.1186/s12870-017-1157-5 29187153 PMC5706331

[B38] Dörnenburg H. (2004). Evaluation of immobilisation effects on metabolic activities and productivity in plant cell processes. *Process Biochem.* 39 1369–1375. 10.1016/S0032-9592(03)00262-0

[B39] Dzier A. Kurkiewicz S. Stêpień K. (2012). Detection and quantitation of a pheomelanin component in melanin pigments using pyrolysis-gas chromatography / tandem mass spectrometry system with multiple reaction monitoring mode. *J. Mass Spectrom.* 2012 242–245. 10.1002/jms.2957 22359335

[B40] Fahad S. Hussain S. Bano A. Saud S. Hassan S. Shan D. (2015). Potential role of phytohormones and plant growth-promoting rhizobacteria in abiotic stresses: consequences for changing environment. *Environ. Sci. Pollut. Res.* 22 4907–4921. 10.1007/s11356-014-3754-2 25369916

[B41] Farag M. A. Zhang H. Ryu C.-M. (2013). Dynamic chemical communication between plants and bacteria through airborne signals: induced resistance by bacterial volatiles. *J. Chem. Ecol.* 39 1007–1018. 10.1007/s10886-013-0317-9 23881442 PMC3738840

[B42] Fekete S. Schappler J. Veuthey J.-L. Guillarme D. (2014). Current and future trends in UHPLC. *TrAC Trends Anal. Chem.* 63 2–13. 10.1016/j.trac.2014.08.007 25391566

[B43] Fernie A. R. Stitt M. (2012). On the discordance of metabolomics with proteomics and transcriptomics: coping with increasing complexity in logic, chemistry and network interactions. *Plant Physiol.* 158 1139–1145. 10.1104/pp.112.193235 22253257 PMC3291261

[B44] Ferreira J. J. del Castillo R. R. Perez-Vega E. Plans M. Simo J. Casanas F. (2012). Sensory changes related to breeding for plant architecture and resistance to viruses and anthracnose in bean market class Fabada (*Phaseolus vulgaris* L.). *Euphytica* 186 687–696. 10.1007/s10681-011-0540-9

[B45] Ferry-Dumazet H. Gil L. Deborde C. Moing A. Bernillon S. Rolin D. (2011). MeRy-B: a web knowledgebase for the storage, visualization, analysis and annotation of plant NMR metabolomic profiles. *BMC Plant Biol.* 11:104. 10.1186/1471-2229-11-104 21668943 PMC3141636

[B46] Finkel O. M. Castrillo G. Herrera Paredes S. Salas González I. Dangl J. L. (2017). Understanding and exploiting plant beneficial microbes. *Curr. Opin. Plant Biol.* 38 155–163. 10.1016/j.pbi.2017.04.018 28622659 PMC5561662

[B47] Fletcher J. S. Kotze H. L. Armitage E. G. Lockyer N. P. Vickerman J. C. (2013). Evaluating the challenges associated with time-of-fight secondary ion mass spectrometry for metabolomics using pure and mixed metabolites. *Metabolomics* 9 535–544. 10.1007/s11306-012-0487-4

[B48] Fujii T. Matsuda S. Tejedor M. L. Esaki T. Sakane I. Mizuno H. (2015). Direct metabolomics for plant cells by live single-cell mass spectrometry direct metabolomics for plant cells by live single-cell mass spectrometry. *Nat. Protoc.* 10 1445–1456. 10.1038/nprot.2015-084 26313480

[B49] Fukushima A. Kusano M. (2013). Recent progress in the development of metabolome databases for plant systems biology. *Front. Plant Sci.* 4:73. 10.3389/fpls.2013.00073 23577015 PMC3616245

[B50] Fukushima A. Kusano M. Redestig H. Arita M. Saito K. (2009). Integrated omics approaches in plant systems biology. *Curr. Opin. Chem. Biol.* 13 532–538. 10.1016/j.cbpa.2009.09.022 19837627

[B51] Gamir J. Flors V. Sanchez-Bel P. (2014). Molecular and physiological stages of priming: how plants prepare for environmental challenges. *Plant Cell Rep.* 33 1935–1949. 10.1007/s00299-014-1665-9 25113544

[B52] Gao X. Chen X. Lin W. Chen S. Lu D. Niu Y. (2013). Bifurcation of *Arabidopsis* NLR immune signaling via Ca2+-dependent protein kinases. *PLOS Pathog.* 9:e1003127. 10.1371/journal.ppat.1003127 23382673 PMC3561149

[B53] Garcia-Fraile P. Menendez E. Rivas R. (2015). Role of bacterial biofertilizers in agriculture and forestry. *AIMS Bioeng.* 2 183–205. 10.3934/bioeng.2015.3.183

[B54] Gomez-Gonzalez S. Ruiz-Jimenez J. Priego-Dapote F. De Castro M. D. L. (2010). Qualitative and quantitative sugar profiling in olive fruits, leaves, and stems by gas chromatography- tandem mass spectrometry (GC-MS / MS) after ultrasound-assisted leaching. *J. Agric. Food Chem.* 58 12292–12299. 10.1021/jf102350s 21058721

[B55] González J. E. Marketon M. M. (2003). Quorum sensing in nitrogen-fixing rhizobia. *Microbiol. Mol. Biol. Rev.* 67 574–592. 10.1128/MMBR.67.4.574 14665677 PMC309046

[B56] Gupta G. Parihar S. S. Ahirwar N. K. Snehi S. K. Singh V. (2015). Plant growth promoting rhizobacteria (PGPR): current and future prospects for development of sustainable agriculture. *J. Microb. Biochem. Technol.* 7 96–102. 10.4172/1948-5948.1000188

[B57] Gust A. A. Brunner F. Nürnberger T. (2010). Biotechnological concepts for improving plant innate immunity. *Curr. Opin. Biotechnol.* 21 204–210. 10.1016/j.copbio.2010.02.004 20181472

[B58] Haas D. Défago G. (2005). Biological control of soil-borne pathogens by fluorescent pseudomonads. *Nat. Rev. Microbiol.* 3 307–319. 10.1038/nrmicro1129 15759041

[B59] Hacquard S. Spaepen S. Garrido-Oter R. Schulze-Lefert P. (2017). Interplay between innate immunity and the plant microbiota. *Annu. Rev. Phytopathol.* 55 565–589. 10.1146/annurev-phyto-080516-035623 28645232

[B60] Haggarty J. Burgess K. E. (2017). Recent advances in liquid and gas chromatography methodology for extending coverage of the metabolome. *Curr. Opin. Biotechnol.* 43 77–85. 10.1016/j.copbio.2016.09.006 27771607

[B61] Haichar F. E. Z. Roncato M.-A. Achouak W. (2012). Stable isotope probing of bacterial community structure and gene expression in the rhizosphere of *Arabidopsis thaliana*. *FEMS Microbiol. Ecol.* 81 291–302. 10.1111/j.1574-6941.2012.01345.x 22385286

[B62] Haichar F. E. Z. Santaella C. Heulin T. Achouak W. (2014). Root exudates mediated interactions belowground. *Soil Biol. Biochem.* 77 69–80. 10.1016/j.soilbio.2014.06.017

[B63] Han S. Li D. Trost E. Mayer K. F. Vlot A. C. Heller W. (2016). Systemic responses of barley to the lactone producing plant beneficial endophyte *Acidovorax radicis* N35. *Front. Plant Sci.* 7:1868. 10.3389/fpls.2016.01868 28018401 PMC5149536

[B64] Hassan R. Shaaban M. I. Abdel Bar F. M. El-Mahdy A. M. Shokralla S. (2016). Quorum sensing inhibiting activity of *Streptomyces coelicoflavus* isolated from soil. *Front. Microbiol.* 7:659. 10.3389/fmicb.2016.00659 27242690 PMC4866617

[B65] Hassan S. Mathesius U. (2012). The role of flavonoids in root-rhizosphere signalling: opportunities and challenges for improving plant-microbe interactions. *J. Exp. Bot.* 63 3429–3444. 10.1093/jxb/err430 22213816

[B66] Hayes R. Ahmed A. Edge T. Zhang H. (2014). Core-shell particles: preparation, fundamentals and applications in high performance liquid chromatography. *J. Chromatogr. A* 1357 36–52. 10.1016/j.chroma.2014.05.010 24856904

[B67] He J. Luo Z. Huang L. He J. Chen Y. Rong X. (2015). Ambient mass spectrometry imaging metabolomics method provides novel insights into the action mechanism of drug candidates. *Anal. Chem.* 87 5372–5379. 10.1021/acs.analchem.5b00680 25874739

[B68] Heil M. Silva Bueno J. C. (2007). Within-plant signaling by volatiles leads to induction and priming of an indirect plant defense in nature. *Proc. Natl. Acad. Sci. U.S.A.* 104 5467–5472. 10.1073/pnas.0610266104 17360371 PMC1838500

[B69] Heinken A. Thiele I. (2015). Systems biology of host-microbe metabolomics. *Wiley Interdiscip. Rev. Syst. Biol. Med.* 7 195–219. 10.1002/wsbm.1301 25929487 PMC5029777

[B70] Helman Y. Chernin L. (2015). Silencing the mob: disrupting quorum sensing as a means to fight plant disease. *Mol. Plant Pathol.* 16 316–329. 10.1111/mpp.12180 25113857 PMC6638422

[B71] Herman M. A. B. Davidson J. K. Smart C. D. (2008). Induction of plant defense gene expression by plant activators and *Pseudomonas syringae* pv. tomato in greenhouse-grown tomatoes. *Phytopathology* 98 1226–1232. 10.1094/PHYTO-98-11-1226 18943412

[B72] Herrgård M. J. Lee B. Portnoy V. Palsson B. Ø (2006). Integrated analysis of regulatory and metabolic networks reveals novel regulatory mechanisms in Saccharomyces cerevisiae. *Genome Res.* 16 627–635. 10.1101/gr.4083206.predict 16606697 PMC1457053

[B73] Heyman H. M. Dubery I. A. (2016). The potential of mass spectrometry imaging in plant metabolomics: a review. *Phytochem. Rev.* 15 297–316. 10.1007/s11101-015-9416-2 28438675

[B74] Hilker M. Schwachtje J. Baier M. Balazadeh S. Bäurle I. Geiselhardt S. (2015). Priming and memory of stress responses in organisms lacking a nervous system. *Biol. Rev.* 49 1118–1133. 10.1111/brv.12215 26289992

[B75] Hird S. J. Lau B. P. Y. Schuhmacher R. Krska R. (2014). Liquid chromatography-mass spectrometry for the determination of chemical contaminants in food. *TrAC - Trends Anal. Chem.* 59 59–72. 10.1016/j.trac.2014.04.005

[B76] Holeski L. M. Jander G. Agrawal A. A. (2012). Transgenerational defense induction and epigenetic inheritance in plants. *Trends Ecol. Evol.* 27 618–626. 10.1016/j.tree.2012.07.011 22940222

[B77] Hong K. W. Koh C. L. Sam C. K. Yin W. F. Chan K. G. (2012). Quorum quenching revisited-from signal decays to signalling confusion. *Sensors* 12 4661–4696. 10.3390/s120404661 22666051 PMC3355433

[B78] Insam H. Seewald M. S. A. (2010). Volatile organic compounds (VOCs) in soils. *Biol. Fertil. Soils* 46 199–213. 10.1007/s00374-010-0442-3

[B79] Iriti M. Faoro F. (2009). Chemical diversity and defence metabolism: how plants cope with pathogens and ozone pollution. *Int. J. Mol. Sci.* 10 3371–3399. 10.3390/ijms10083371 20111684 PMC2812827

[B80] Jin Y. Deng Y. Yue J. Zhao Y. Yu W. Liu Z. (2015). Significant improvements in the characterization of volatile compound profiles in squid using simultaneous distillation-extraction and GC × GC-TOFMS. *CyTA J. Food* 13 434–444. 10.1080/19476337.2014.997798

[B81] Johns N. I. Blazejewski T. Gomes A. L. C. Wang H. H. (2016). Principles for designing synthetic microbial communities. *Curr. Opin. Microbiol.* 31 146–153. 10.1016/j.mib.2016.03.010 27084981 PMC4899134

[B82] Jones J. D. G. Dangl J. L. (2006). The plant immune system. *Nature* 444 323–329. 10.1038/nature05286 17108957

[B83] Jorge T. F. Rodrigues A. Caldana C. Schmidt R. van Dongen J. T. Thomas-Oates J. (2016). Mass spectrometry-based plant metabolomics: metabolites responses to abiotic stresses. *Mass Spectrom. Rev.* 35 620–649. 10.1002/mas 25589422

[B84] Kai M. Effmert U. Piechulla B. (2016). Bacterial-plant-interactions: approaches to unravel the biological function of bacterial volatiles in the rhizosphere. *Front. Microbiol.* 7:108. 10.3389/fmicb.2016.00108 26903987 PMC4746483

[B85] Kai M. Haustein M. Molina F. Petri A. Scholz B. Piechulla B. (2009). Bacterial volatiles and their action potential. *Appl. Microbiol. Biotechnol.* 81 1001–1012. 10.1007/s00253-008-1760-3 19020812

[B86] Kai M. Uta E. V. Berg G. Piechulla B. (2007). Volatiles of bacterial antagonists inhibit mycelial growth of the plant pathogen *Rhizoctonia solani*. *Arch. Microbiol.* 187 351–360. 10.1007/s00203-006-0199-0 17180381

[B87] Kanchiswamy C. N. Malnoy M. Maffei M. E. (2015). Chemical diversity of microbial volatiles and their potential for plant growth and productivity. *Front. Plant Sci.* 6:151. 10.3389/fpls.2015.00151 25821453 PMC4358370

[B88] Kloepper J. W. Ryu C.-M. Zhang S. (2004). Induced systemic resistance and promotion of plant growth by *Bacillus* spp. *Phytopathology* 94 1259–1266. 10.1094/PHYTO.2004.94.11.1259 18944464

[B89] Koornneef A. Pieterse C. M. J. (2008). Cross talk in defense signaling1. *Plant Physiol.* 146 839–844. 10.1104/pp.107.112029 18316638 PMC2259093

[B90] Kravchenko L. V. Azarova T. S. Shaposhnikov A. I. Makarova N. M. Tikhonovich I. A. (2003). Root exudates of tomato plants and their effect on the growth and antifungal activity of *Pseudomonas* strains. *Microbiology* 72 37–41. 10.1023/A:1022269821379 12698791

[B91] Kuich P. H. J. L. Hoofmann N. Kempa S. (2015). Maui-VIA: a user-friendly software for visual identification, alignment, correction, and quantification of gas chromatography– mass spectrometry data. *Front. Bioeng. Biotechnol.* 2:84. 10.3389/fbioe.2014.00084 25654076 PMC4301187

[B92] Lei Z. Huhman D. V. Sumner L. W. (2011). Mass spectrometry strategies in metabolomics. *J. Biol. Chem.* 286 25435–25442. 10.1074/jbc.R111.238691 21632543 PMC3138266

[B93] Li X. Zhang T. Wang X. Hua K. Zhao L. Han Z. (2013). The composition of root exudates from two different resistant peanut cultivars and their effects on the growth of soil-borne pathogen. *Int. J. Biol. Sci.* 9 164–173. 10.7150/ijbs.5579 23412138 PMC3572399

[B94] Liu W. Liu J. Ning Y. Ding B. Wang X. Wang Z. (2013). Recent progress in understanding PAMP- and effector-triggered immunity against the rice blast fungus *Magnaporthe oryzae*. *Mol. Plant* 6 605–620. 10.1093/mp/sst015 23340743

[B95] Lloyd N. Johnson D. L. Herderich M. J. (2015). Metabolomics approaches for resolving and harnessing chemical diversity in grapes, yeast and wine. *Aust. J. Grape Wine Res.* 21 723–740. 10.1111/ajgw.12202

[B96] Lohitha S. R. Bhaskara R. B. V. Sivaprasad Y. Prathyusha M. Sujitha A. Krishna T. (2016). Molecular characterization and antagonistic potential of phenazine-1-carboxylic acid producing *Pseudomonas fluorescens* isolates from economically important crops in South India. *Int. J. Clin. Biol. Sci.* 1 30–40.

[B97] Luna E. Bruce T. J. A. Roberts M. R. Flors V. Ton J. (2012). Next-generation systemic acquired resistance. *Plant Physiol.* 158 844–853. 10.1104/pp.111.187468 22147520 PMC3271772

[B98] Madala N. E. Tugizimana F. Steenkamp P. (2014). Development and optimization of an UPLC-QTOF-MS/MS method based on an in-source collision induced dissociation approach for comprehensive discrimination of chlorogenic acids isomers from Momordica plant species. *J. Anal. Methods Chem.* 2014 1–7. 10.1155/2014/650879 25295221 PMC4177087

[B99] Mandal R. Kathiria P. Psychogios N. Bouatra S. Krishnamurthy R. Wishart D. (2012). Progeny of tobacco mosaic virus-infected *Nicotiana tabacum* plants exhibit trans-generational changes in metabolic profiles. *Biocatal. Agric. Biotechnol.* 1 115–123. 10.1016/j.bcab.2012.01.004

[B100] Massalha H. Korenblum E. Tholl D. Aharoni A. (2017). Small molecules below-ground: the role of specialized metabolites in the rhizosphere. *Plant J.* 90 788–807. 10.1111/tpj.13543 28333395

[B101] Mauch-Mani B. Baccelli I. Luna E. Flors V. (2017). Defense priming: an adaptive part of induced resistance. *Annu. Rev. Plant Biol.* 68 485–512. 10.1146/annurev-arplant-042916-041132 28226238

[B102] Mercado-Blanco J. Bakker P. A. (2007). Interactions between plants and beneficial *Pseudomonas* spp.: exploiting bacterial traits for crop protection. *Antonie Van Leeuwenhoek* 92 367–389. 10.1007/s10482-007-9167-1 17588129

[B103] Mhlongo M. I. Piater L. A. Steenkamp P. A. Madala N. E. Dubery I. A. (2016a). Phenylpropanoid defences in *Nicotiana tabacum* cells: overlapping metabolomes indicate common aspects to priming responses induced by lipopolysaccharides. *PLOS ONE* 11:e0151350. 10.1371/journal.pone.0151350 26978774 PMC4792386

[B104] Mhlongo M. I. Steenkamp P. A. Piater L. A. Madala N. E. Dubery I. A. (2016b). Profiling of altered metabolomic states in *Nicotiana tabacum* cells induced by priming agents. *Front. Plant Sci.* 7:1527. 10.3389/fpls.2016.01527 27803705 PMC5068090

[B105] Mhlongo M. I. Tugizimana F. Piater L. A. Steenkamp P. A. Madala N. E. Dubery I. A. (2017). Untargeted metabolomics analysis reveals dynamic changes in azelaic acid- and salicylic acid derivatives in LPS-treated *Nicotiana tabacum* cells. *Biochem. Biophys. Res. Commun.* 482 1498–1503. 10.1016/j.bbrc.2016.12.063 27956183

[B106] Mommer L. Kirkegaard J. van Ruijven J. (2016). Root– root interactions: towards a rhizosphere framework. *Trends Plant Sci.* 21 209–217. 10.1016/j.tplants.2016.01.009 26832947

[B107] Munné-Bosch S. Alegre L. (2013). Cross-stress tolerance and stress “memory” in plants: an integrated view. *Environ. Exp. Bot.* 94 1–2. 10.1016/j.envexpbot.2013.02.002

[B108] Naseem M. Dandekar T. (2012). The role of auxin-cytokinin antagonism in plant-pathogen interactions. *PLOS Pathog.* 8:e1003026. 10.1371/journal.ppat.1003026 23209407 PMC3510258

[B109] Naz S. (2014). Analytical protocols based on LC–MS, GC–MS and CE–MS for nontargeted metabolomics of biological tissues. *Bioanalysis* 6 1657–1677. 10.4155/BIO.14.119 25077626

[B110] Ncube E. N. Mhlongo M. I. Piater L. A. Steenkamp P. A. Dubery I. A. Madala N. E. (2014). Analyses of chlorogenic acids and related cinnamic acid derivatives from *Nicotiana tabacum* tissues with the aid of UPLC-QTOF-MS / MS based on the in-source collision-induced dissociation method. *Chem. Cent. J.* 8 66. 10.1186/s13065-014-0066-z 25426160 PMC4242998

[B111] Nizkorodov S. A. Laskin J. Laskin A. (2011). Molecular chemistry of organic aerosols through the application of high resolution mass spectrometry. *Phys. Chem. Chem. Phys.* 13 3612–3629. 10.1039/c0cp02032j 21206953

[B112] Ofaim S. Ofek-Lalzar M. Sela N. Jinag J. Kashi Y. Minz D. (2017). Analysis of microbial functions in the rhizosphere using a metabolomic-network based framework for metagenomics interpretation. *Front. Microbiol.* 8:1606. 10.3389/fmicb.2017.01606 28878756 PMC5572346

[B113] Okazaki Y. Saito K. (2012). Recent advances of metabolomics in plant biotechnology. *Plant Biotechnol. Rep.* 6 1–15. 10.1007/s11816-011-0191-2 22308170 PMC3262138

[B114] Ongena M. Duby F. Jourdan E. Beaudry T. Jadin V. Dommes J. (2005a). *Bacillus subtilis* M4 decreases plant susceptibility towards fungal pathogens by increasing host resistance associated with differential gene expression. *Appl. Microbiol. Biotechnol.* 67 692–698. 10.1007/s00253-004-1741-0 15578181

[B115] Ongena M. Jacques P. Touré Y. Destain J. Jabrane A. (2005b). Involvement of fengycin-type lipopeptides in the multifaceted biocontrol potential of *Bacillus subtilis*. *Appl. Microbiol. Biotechnol.* 69 29–38. 10.1007/s00253-005-1940-3 15742166

[B116] Ongena M. Jourdan E. Adam A. Paquot M. Brans A. Joris B. (2007). Surfactin and fengycin lipopeptides of *Bacillus subtilis* as elicitors of induced systemic resistance in plants. *Environ. Microbiol.* 9 1084–1090. 10.1111/j.1462-2920.2006.01202.x 17359279

[B117] Onjiko R. M. Moody S. A. Nemes P. (2015). Single-cell mass spectrometry reveals small molecules that affect cell fates in the 16-cell embryo. *Proc. Natl. Acad. Sci. U.S.A.* 112 6545–6550. 10.1073/pnas.1423682112 25941375 PMC4450407

[B118] Park J. Jeong H. Kang B. Kim S. J. Park S. Y. (2015). Multi-dimensional TOF-SIMS analysis for effective profiling of disease-related ions from the tissue surface. *Sci. Rep.* 5:11077. 10.1038/srep11077 26046669 PMC4457153

[B119] Pastor V. Balmer A. Gamir J. Flors V. Mauch-Mani B. (2014). Preparing to fight back: generation and storage of priming compounds. *Front. Plant Sci.* 5:295. 10.3389/fpls.2014.00295 25009546 PMC4068018

[B120] Pastor V. Luna E. Mauch-Mani B. Ton J. Flors V. (2012). Primed plants do not forget. *Environ. Exp. Bot.* 94 46–56. 10.1016/j.envexpbot.2012.02.013

[B121] Patil S. Bheemaraddi M. C. Shivannavar C. T. (2014). Biocontrol activity of siderophore producing *Bacillus subtilis* CTS-G24 against wilt and dry root rot causing fungi in chickpea. *J. Agric. Vet. Sci.* 7 63–68.

[B122] Pattus F. Abdallah A. (2000). Siderophores and iron-transport in microorganisms. *J. Chin. Chem. Soc.* 47 1–20. 10.1002/jccs.200000001

[B123] Pickett J. A. Birkett M. A. Bruce T. J. A. Chamberlain K. Gordon-Weeks R. Matthes M. C. (2007). Developments in aspects of ecological phytochemistry: the role of cis-jasmone in inducible defence systems in plants. *Phytochemistry* 68 2937–2945. 10.1016/j.phytochem.2007.09.025 18023830

[B124] Pieterse C. M. J. (2012). Prime time for transgenerational defense. *Plant Physiol.* 158:545. 10.1104/pp.112.900430 22308198 PMC3271748

[B125] Pieterse C. M. J. Leon-Reyes A. Van der Ent S. Van Wees S. C. M. (2009). Networking by small-molecule hormones in plant immunity. *Nat. Chem. Biol.* 5 308–316. 10.1038/nchembio.164 19377457

[B126] Pineda A. Kaplan I. Bezemer T. M. (2017). Steering soil microbiomes to suppress aboveground insect pests. *Trends Plant Sci.* 22 770–778. 10.1016/j.tplants.2017.07.002 28757147

[B127] Pineda A. Zheng S.-J. van Loon J. J. A. Pieterse C. M. J. Dicke M. (2010). Helping plants to deal with insects: the role of beneficial soil-borne microbes. *Trends Plant Sci.* 15 507–514. 10.1016/j.tplants.2010.05.007 20542720

[B128] Planchamp C. Glauser G. Mauch-Mani B. (2014). Root inoculation with *Pseudomonas putida* KT2440 induces transcriptional and metabolic changes and systemic resistance in maize plants. *Front. Plant Sci.* 5:719. 10.3389/fpls.2014.00719 25628626 PMC4292437

[B129] Po-Wen C. Singh P. Zimmerli L. (2013). Priming of the *Arabidopsis* pattern-triggered immunity response upon infection by necrotrophic *Pectobacterium carotovorum* bacteria. *Mol. Plant Pathol.* 14 58–70. 10.1111/j.1364-3703.2012.00827.x 22947164 PMC6638802

[B130] Preti R. (2016). Core-shell columns in high-performance liquid chromatography: Food analysis applications. *Int. J. Anal. Chem.* 2016:3189724. 10.1155/2016/3189724 27143972 PMC4842074

[B131] Ramautar R. Somsen G. W. de Jong G. J. (2015). CE-MS for metabolomics: developments and applications in the period 2012–2014. *Electrophoresis* 36 212–224. 10.1002/elps.201400388 25287884

[B132] Ramautar R. Somsen G. W. de Jong G. J. (2016). CE–MS for metabolomics: developments and applications in the period 2014–2016. *Electrophoresis* 38 1–13. 10.1002/elps.201600370 27718257 PMC5248609

[B133] Ramos-Solano B. Algar E. Gutierrez-Mañero F. J. Bonilla A. Lucas J. A. García-Seco D. (2015). Bacterial bioeffectors delay postharvest fungal growth and modify total phenolics, flavonoids and anthocyanins in blackberries. *LWT Food Sci. Technol.* 61 437–443. 10.1016/j.lwt.2014.11.051

[B134] Rao W. Pan N. Yang Z. (2016). Applications of the single-probe: mass spectrometry imaging and single cell analysis under ambient conditions. *J. Vis. Exp.* 112:e53911. 10.3791/53911 27341402 PMC4924803

[B135] Rochfort S. (2005). Metabolomics reviewed: a new “omics” platform technology for systems biology and implications for natural products research. *J. Nat. Prod.* 68 1813–1820. 10.1021/np050255w 16378385

[B136] Romero D. de Vicente A. Rakotoaly R. H. Dufour S. E. Veening J.-W. Arrebola E. (2007). The iturin and fengycin families of lipopeptides are key factors in antagonism of *Bacillus subtilis* toward *Podosphaera fusca*. *Mol. Plant Microbe Interact.* 20 430–440. 10.1094/MPMI-20-4-0430 17427813

[B137] Rosier A. Bishnoi U. Lakshmanan V. (2016). A perspective on inter-kingdom signaling in plant-beneficial microbe interactions. *Plant Mol. Biol.* 90 537–548. 10.1007/s11103-016-0433-3 26792782

[B138] Rothballer M. Uhl J. Kunze J. Schmitt-Kopplin P. Hartmann A. (2018). Detection of the bacterial quorum-sensing signaling molecules N-acyl-homoserine lactones (HSL) and N-acyl-homoserine (HS) with an enzyme-linked immunosorbent assay (ELISA) and via ultrahigh-performance liquid chromatography coupled to mass spectrometry (UHPLC-MS). *Methods Mol. Biol.* 1673 61–72. 29130164 10.1007/978-1-4939-7309-5_5

[B139] Ryu C. Farag M. A. Hu C. Reddy M. S. Kloepper J. W. Pare P. W. (2004). Bacterial volatiles induced resistance in *Arabidopsis*. *Plant Physiol.* 134 1017–1026. 10.1104/pp.103.026583 14976231 PMC389924

[B140] Ryu C.-M. Farag M. A. Hu C.-H. Reddy M. S. Wei H.-X. Pare P. W. (2003). Bacterial volatiles promote growth in Arabidopsis. *Proc. Natl. Acad. Sci. U.S.A.* 100 4927–4932. 10.1073/pnas.0730845100 12684534 PMC153657

[B141] Saha D. Purkayastha G. D. Ghosh A. Isha M. Saha A. (2012). Isolation and characterization of two new *Bacillus subtilis* strains from the rhizosphere of eggplant as potential biocontrol agents. *J. Plant Pathol.* 94 109–118. 10.4454/jpp.fa.2012.020

[B142] Sakurai N. Ara T. Enomoto M. Motegi T. Kurabayashi A. Iijima Y. (2014). Tools and databases of the KOMICS web portal for preprocessing, mining, and dissemination of metabolomics data. *Biomed. Res. Int.* 2014:194812. 10.1155/2014/194812 24949426 PMC4052814

[B143] Sakurai T. Yamnda Y. Sawanda Y. Matsuda F. Akiyima K. Shinozaki K. (2013). PRIMe Update: innovative content for plant metabolomics and integration of gene expression and metabolite accumulation. *Plant Cell Physiol.* 54:e5. 10.1093/pcp/pcs184 23292601 PMC3583026

[B144] Sanabria N. M. Huang J. Dubery I. A. (2009). Self/non-self perception in plants in innate immunity and defense. *Self Nonself* 1 40–54. 10.4161/self.1.1.10442 21559176 PMC3091600

[B145] Sanchez A. C. Friedlander G. Fekete S. Anspach J. Guillarme D. Chitty M. (2013). Pushing the performance limits of reversed-phase ultra high performance liquid chromatography with 1.3μm core-shell particles. *J. Chromatogr. A* 1311 90–97. 10.1016/j.chroma.2013.08.065 24016720

[B146] Saraf M. Pandya U. Thakkar A. (2005). Use of plant growth-promoting bacteria for biocontrol of plant diseases: principles, mechanisms of action, and future prospects. *Appl. Environ. Microbiol.* 71 4951–4959. 10.1128/AEM.71.9.4951 16151072 PMC1214602

[B147] Saraf M. Pandya U. Thakkar A. (2014). Role of allelochemicals in plant growth promoting rhizobacteria for biocontrol of phytopathogens. *Microbiol. Res.* 169 18–29. 10.1016/j.micres.2013.08.009 24176815

[B148] Sasse J. Martinoia E. Northen T. (2017). Feed your friends: do plant exudates shape the root microbiome? *Trends Plant Sci.* 23 25–41. 10.1016/j.tplants.2017.09.003 29050989

[B149] Schenk S. T. Hernández-reyes C. Samans B. Stein E. Neumann C. Schikora M. (2014). N-acyl-homoserine lactone primes plants for cell wall reinforcement and induces resistance to bacterial pathogens via the salicylic acid / oxylipin pathway. *Plant Cell* 26 2708–2723. 10.1105/tpc.114.126763 24963057 PMC4114961

[B150] Schikora A. (2016). Beneficial effects of bacteria-plant communication based on quorum sensing molecules of the N -acyl homoserine lactone group. *Plant Methods* 90 605–612. 10.1007/s11103-016-0457-8 26898296

[B151] Schuhegger R. Ihring A. Gantner S. Bahnweg G. Knappe C. Vogg G. (2006). Induction of systemic resistance in tomato by N-acyl-L-homoserine lactone-producing rhizosphere bacteria. *Plant Cell Environ.* 29 909–918. 10.1111/j.1365-3040.2005.01471.x 17087474

[B152] Schwamborn K. (2012). Imaging mass spectrometry in biomarker discovery and validation. *J. Proteomics* 75 4990–4998. 10.1016/j.jprot.2012.06.015 22749859

[B153] Segarra G. Van der Ent S. Trillas I. Pieterse C. M. J. (2009). MYB72 a node of convergence in induced systemic resistance triggered by a fungal and a bacterial beneficial microbe. *Plant Biol.* 11 90–96. 10.1111/j.1438-8677.2008.00162.x 19121118

[B154] Slaughter A. Daniel X. Flors V. Luna E. Hohn B. Mauch-Mani B. (2012). Descendants of primed *Arabidopsis* plants exhibit resistance to biotic stress. *Plant Physiol.* 158 835–843. 10.1104/pp.111.191593 22209872 PMC3271771

[B155] Song G. C. Ryu C.-M. (2013). Two volatile organic compounds trigger plant self-defense against a bacterial pathogen and a sucking insect in cucumber under open field conditions. *Int. J. Mol. Sci.* 14 9803–9819. 10.3390/ijms14059803 23698768 PMC3676814

[B156] Song Y. Y. Cao M. Xie L. J. Liang X. T. Zeng R. Sen Su Y. J. (2011). Induction of DIMBOA accumulation and systemic defense responses as a mechanism of enhanced resistance of mycorrhizal corn (*Zea mays* L.) to sheath blight. *Mycorrhiza* 21 721–731. 10.1007/s00572-011-0380-4 21484338

[B157] Steinbeck C. Conesa P. Haug K. Mahendraker T. Williams M. Maguire E. (2012). MetaboLights: towards a new COSMOS of metabolomics data management. *Metabolomics* 8 757–760. 10.1007/s11306-012-0462-0 23060735 PMC3465651

[B158] Tank N. Rajendran N. Patel B. Saraf M. (2012). Evaluation and biochemical characterization of a distinctive pyoverdin from a *Pseudomonas* isolated from Chickpea rhizosphere. *Braz. J. Microbiol.* 1 639–648. 10.1590/S1517-83822012000200028 24031875 PMC3768837

[B159] Tanou G. Fotopoulos V. Molassiotis A. (2012). Priming against environmental challenges and proteomics in plants: update and agricultural perspectives. *Front. Plant Sci.* 3:216. 10.3389/fpls.2012.00216 22973291 PMC3438484

[B160] Tenenboim H. Brotman Y. (2016). Omic relief for the biotically stressed: metabolomics of plant biotic interactions. *Trends Plant Sci.* 21 781–791. 10.1016/j.tplants.2016.04.009 27185334

[B161] Thakur M. Sohal B. S. (2013). Role of elicitors in inducing resistance in plants against pathogen infection: a review. *ISRN Biochem.* 2013:762412. 10.1155/2013/762412 25969762 PMC4393000

[B162] Thorpe A. S. Thelen G. C. Diaconu A. Callaway R. M. (2009). Root exudate is allelopathic in invaded community but not in native community: field evidence for the novel weapons hypothesis. *J. Ecol.* 97 641–645. 10.1111/j.1365-2745.2009.01520.x 21882071

[B163] Toussaint J. P. Smith F. A. Smith S. E. (2007). Arbuscular mycorrhizal fungi can induce the production of phytochemicals in sweet basil irrespective of phosphorus nutrition. *Mycorrhiza* 17 291–297. 10.1007/s00572-006-0104-3 17273856

[B164] Trivedi D. K. Iles R. K. (2012). The application of SIMCA P+ in shotgun metabolomics analysis of ZIC-HILIC-MS spectra of human urine- experience with the Shimadzu IT-TOF and profiling solutions data extraction software. *J. Chromatogr. Sep. Tech.* 3 2–5. 10.4172/2157-7064.1000145

[B165] Tugizimana F. Piater L. A. Dubery I. A. (2013). Plant metabolomics: a new frontier in phytochemical analysis. *S. Afr. J. Sci.* 109 18–20. 10.1590/sajs.2013/20120005

[B166] Tugizimana F. Steenkamp P. A. Piater L. A. Dubery I. A. (2014). Multi-platform metabolomic analyses of ergosterol-induced dynamic changes in *Nicotiana tabacum* cells. *PLOS ONE* 9:e87846. 10.1371/journal.pone.0087846 24498209 PMC3909234

[B167] Tyc O. Zweers H. de Boer W. Gardeva P. (2015). Volatiles in inter-specific bacterial interactions. *Front. Microbiol.* 6:1412 10.3389/fmicb.2015.01412 PMC4683202 26733959

[B168] Uhrig R. G. Labandera A.-M. Moorhead G. B. (2013). *Arabidopsis* PPP family of serine/threonine protein phosphatases: many targets but few engines. *Trends Plant Sci.* 18 505–513. 10.1016/j.tplants.2013.05.004 23790269

[B169] Urio R. P. Masini J. C. (2015). Evaluation of monolithic and core-shell columns for separation of triazine herbicides by reversed phase high performance liquid chromatography. *J. Braz. Chem. Soc.* 26 2331–2338. 10.5935/0103-5053.20150227 25281136

[B170] Vacheron J. Desbrosses G. Bouffaud M.-L. Touraine B. Moënne-Loccoz Y. Muller D. (2013). Plant growth-promoting rhizobacteria and root system functioning. *Front. Plant Sci.* 4:356. 10.3389/fpls.2013.00356 24062756 PMC3775148

[B171] Van Dam N. M. Bouwmeester H. J. (2016). Metabolomics in the rhizosphere: tapping into belowground chemical communication. *Trends Plant Sci.* 21 256–265. 10.1016/j.tplants.2016.01.008 26832948

[B172] van de Mortel J. E. Vos R. C. H. De Dekkers E. Pineda A. Guillod L. Bouwmeester K. (2012). Metabolic and transcriptomic changes induced in Arabidopsis by the rhizobacterium *Pseudomonas*. *Plant Physiol.* 160 2173–2188. 10.1104/pp.112.207324 23073694 PMC3510139

[B173] Van Wees S. C. M. Van der Ent S. Pieterse C. M. J. (2008). Plant immune responses triggered by beneficial microbes. *Curr. Opin. Plant Biol.* 11 443–448. 10.1016/j.pbi.2008.05.005 18585955

[B174] Venturi V. Keel C. (2016). Signaling in the rhizosphere. *Trends Plant Sci.* 21 187–198. 10.1016/j.tplants.2016.01.005 26832945

[B175] Verhage A. van Wees S. C. M. Pieterse C. M. J. (2010). Plant immunity: it’s the hormones talking, but what do they say? *Plant Physiol.* 154 536–540. 10.1104/pp.110.161570 20921180 PMC2949039

[B176] Verhagen B. W. M. Trotel-Aziz P. Couderchet M. Höfte M. Aziz A. (2010). Pseudomonas spp.-induced systemic resistance to *Botrytis cinerea* is associated with induction and priming of defence responses in grapevine. *J. Exp. Bot.* 61 249–260. 10.1093/jxb/erp295 19812243

[B177] Villas-Bôas S. G. Smart K. F. Sivakumaran S. Lane G. A. (2011). Alkylation or silylation for analysis of amino and. *Metabolites* 1 3–20. 10.3390/metabo1010003 24957242 PMC4012512

[B178] Vos I. A. Pieterse C. M. J. van Wees S. C. M. (2013). Costs and benefits of hormone-regulated plant defences. *Plant Pathol.* 62 43–55. 10.1111/ppa.12105

[B179] Walter T. H. Andrews R. W. (2014). Recent innovations in UHPLC columns and instrumentation. *TrAC Trends Anal. Chem.* 63 14–20. 10.1016/j.trac.2014.07.016

[B180] Wei J. Kang L. (2011). Roles of ( Z ) -3-hexenol in plant-insect interactions. *Plant Signal. Behav.* 6 369–371. 10.4161/psb.6.3.14452 21346418 PMC3142417

[B181] Wei-wei L. I. U. Wei M. U. Bing-Yu Z. H. U. You-Chen D. U. Feng L. I. U. (2008). Antagonistic activities of volatiles from four strains of *Bacillus* spp. and *Paenibacillus* spp. against soil-borne plant pathogens. *Agric. Sci. China* 7 1104–1114. 10.1016/S1671-2927(08)60153-4

[B182] West C. Elfakir C. Lafosse M. (2010). Porous graphitic carbon: a versatile stationary phase for liquid chromatography. *J. Chromatogr. A* 1217 3201–3216. 10.1016/j.chroma.2009.09.052 19811787

[B183] Wheatcraft D. R. A. Liu X. Hummon A. B. (2014). Sample preparation strategies for mass spectrometry imaging of 3D cell culture models. *J. Vis. Exp.* 94:e52313. 10.3791/52313 25549242 PMC4396945

[B184] Worley B. Powers R. (2013). Multivariate analysis in metabolomics. *Curr. Metab.* 1 92–107. 10.2174/2213235X11301010092 26078916 PMC4465187

[B185] Yamaguchi Y. Huffaker A. Bryan A. C. Tax F. E. Ryan C. A. (2010). PEPR2 Is a second receptor for the pep1 and pep2 peptides and contributes to defense responses in *Arabidopsis*. *Plant Cell* 22 508–522. 10.1105/tpc.109.068874 20179141 PMC2845411

[B186] Yang Y. Wang N. Guo X. Zhang Y. Ye B. (2017). Comparative analysis of bacterial community structure in the rhizosphere of maize by high throughput pyrosequencing. *PLOS ONE* 12:e178425. 10.1371/journal.pone.0178425 28542542 PMC5444823

[B187] Yi H.-S. Yang J. W. Ryu C.-M. (2013). ISR meets SAR outside: additive action of the endophyte *Bacillus pumilus* INR7 and the chemical inducer, benzothiadiazole, on induced resistance against bacterial spot in field-grown pepper. *Front. Plant Sci.* 4:122. 10.3389/fpls.2013.00122 23717313 PMC3653112

[B188] You L. Zhang B. Tang Y. J. (2014). Application of stable isotope-assisted metabolomics for cell metabolism studies. *Metabolites* 4 142–165. 10.3390/metabo4020142 24957020 PMC4101500

[B189] Zhang J. Zhou J.-M. (2010). Plant immunity triggered by microbial molecular signatures. *Mol. Plant* 3 783–793. 10.1093/mp/ssq035 20713980

[B190] Zhang N. Wang D. Liu Y. (2014). Effects of different plant root exudates and their organic acid components on chemotaxis, biofilm formation and colonization by beneficial rhizosphere-associated bacterial strains. *Plant Soil* 374 689–700. 10.1007/s11104-013-1915-6

[B191] Zhang X. Zhang R. Gao J. Wang X. Fan F. Ma X. (2017). Thirty-one years of rice-rice-green manure rotations shape the rhizosphere microbial community and enrich beneficial bacteria. *Soil Biol. Biochem.* 104 208–217. 10.1016/j.soilbio.2016.10.023

[B192] Zipfel C. (2008). Pattern-recognition receptors in plant innate immunity. *Curr. Opin. Immunol.* 20 10–16. 10.1016/j.coi.2007.11.003 18206360

